# Bioderived Laser-Induced Graphene for Sensors and Supercapacitors

**DOI:** 10.1021/acsami.3c07687

**Published:** 2023-07-20

**Authors:** Anna Chiara Bressi, Alexander Dallinger, Yulia Steksova, Francesco Greco

**Affiliations:** † The Biorobotics Institute, Scuola Superiore Sant’Anna, Viale R. Piaggio 34, 56025 Pontedera, Italy; § Department of Excellence in Robotics & AI, Scuola Superiore Sant’Anna, Piazza Martiri della Libertà 33, 56127 Pisa, Italy; ‡ Institute of Solid State Physics, NAWI Graz, Graz University of Technology, Petergasse 16, Graz 8010, Austria; ⊥ Interdisciplinary Center on Sustainability and Climate, Scuola Superiore Sant’Anna, Piazza Martiri della Libertà 33, 56127 Pisa, Italy

**Keywords:** laser-induced graphene, bioderived, green, transient, electronics, sensing, bioderived

## Abstract

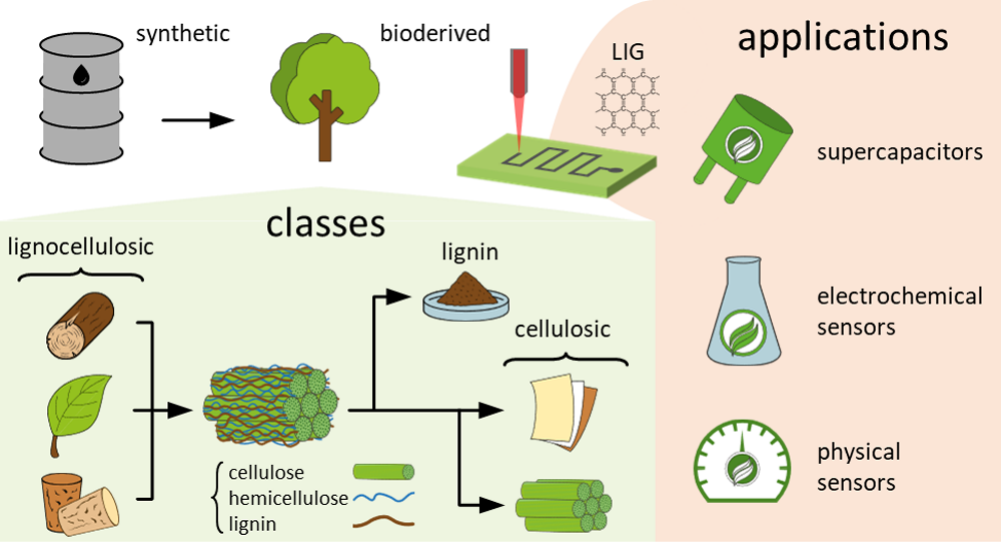

The maskless and chemical-free conversion and patterning of synthetic polymer precursors into laser-induced graphene (LIG) via laser-induced pyrolysis is a relatively new but growing field. Bioderived precursors from lignocellulosic materials can also be converted to LIG, opening a path to sustainable and environmentally friendly applications. This review is designed as a starting point for researchers who are not familiar with LIG and/or who wish to switch to sustainable bioderived precursors for their applications. Bioderived precursors are described, and their performances (mainly crystallinity and sheet resistance of the obtained LIG) are compared. The three main fields of application are reviewed: supercapacitors and electrochemical and physical sensors. The key advantages and disadvantages of each precursor for each application are discussed and compared to those of a benchmark of polymer-derived LIG. LIG from bioderived precursors can match, or even outperform, its synthetic analogue and represents a viable and sometimes better alternative, also considering its low cost and biodegradability.

## 1 Introduction

**Figure 1:**
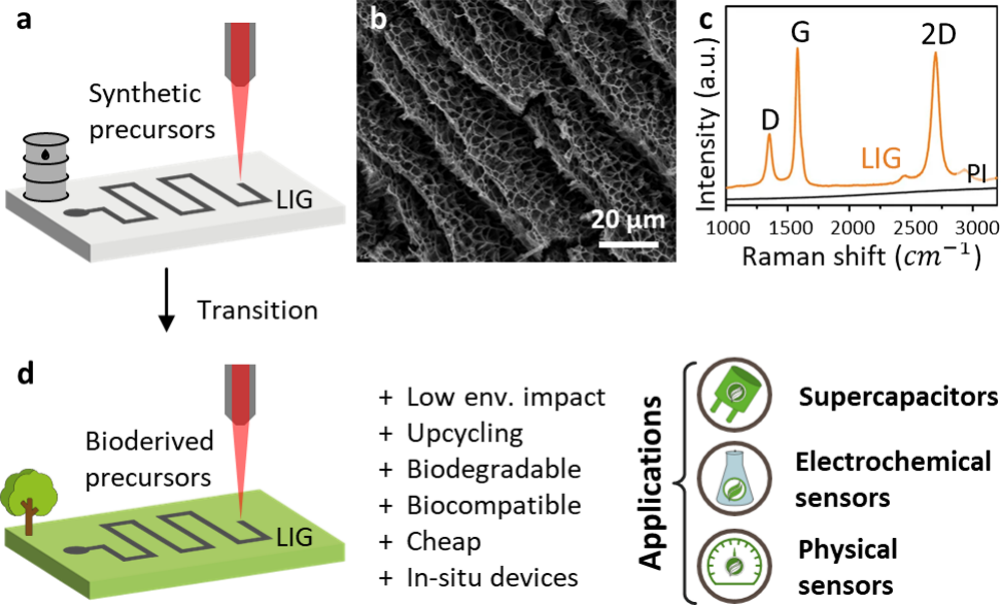
(a) Schematic illustration of the LIG production process from commercial polymers. (b) SEM image of the porous LIG structure. Reprinted with permission from ref (14). Available under a CC BY 4.0 license. (c) Raman spectrum of PI and LIG, showing the three characteristic peaks (D, G, and 2D). Reprinted with permission from ref (1). Copyright 2014 Springer Nature. (d) Advantages and main applications of LIG obtained from bioderived precursors.

New approaches to graphene synthesis and patterning toward applications in devices are being researched, especially the aspects related to environmental and economic sustainability. In 2014, Lin et al.^1^ reported a new fabrication process for graphenic materials: polymeric precursors with high content of aromatic carbons and high thermal stability (e.g., polyimide (PI)) can be converted into a 3D porous carbon structure composed of disordered graphene sheets when irradiated by an IR laser. The transformation happens through a fast photochemical and thermal pyrolytic process, resulting in the so-called laser-induced graphene (LIG). The production of LIG is based on a single-step, scalable, and fast process, not involving the use of chemicals, operated in air with simple - and relatively cheap - laser-scribing equipment, normally employed in workshops for laser cutting/engraving of materials (Figure 1a). The laser scribing allows to directly synthesize and pattern customized LIG conductive tracks on insulating substrates without using masks.^2^ Extremely high temperatures and pressures (>2400 K, ≈3 GPa) are obtained at the polymer surface, where the laser spot is focused. This causes the decomposition of the polymer precursor and an explosive release of gases,^3^ giving origin to the unique, porous, and inhomogeneous structure of LIG (Figure 1b).^4^ The defects are both at the micro level, due to the complex 3D porous structure of 2D graphene layers,^4^ and at the nano level, with defects appearing as pentagons and heptagons in the otherwise hexagonal lattice.^1^ LIG has the typically high thermal and electrical conductivity of graphene,^5^ large surface area,^6^ elastic modulus,^7^ and high stability against corrosion.^8^ The possibility of simultaneous synthesis and patterning of graphene in a single laser scribing step makes LIG an attractive and practical method for various application fields, which include, among others: supercapacitors (SC),^9^ electrochemical sensors (ES),^10^ physical sensors (PS),^11^ environmental sensors,^12^ actuators,^13,14^ and wearable flexible electronics.^2,15^ The resolution of the scribing process can be adapted to the chosen application and depends on the laser source used (UV, visible, or IR). UV and visible lasers can allow for the scribing of smaller, high-resolution features (down to around 5–10 μm and 6–12 μm width of a single scribed line, respectively) compared to the more often used IR lasers (down to around 100 μm).^4,16−18^ Another significant advantage for LIG application is the tunability of the composition and morphology of LIG and, in turn, of its electrical, mechanical, and surface properties, depending on several variables.

A few years after the first report on LIG from synthetic precursors, it was proven that LIG could be directly scribed also on bioderived precursors, such as bread, potato skins, or coconut shells.^19,20^ This shifted the interest from synthetic to bioderived LIG with a focus on the conversion mechanism and on new applications made possible by the use of bioderived or biodegradable precursors (Figure 1d). An advantage of bioderived precursors is the mitigation of the carbon footprint and upcycling of waste materials, following the model of the circular economy. Another interesting opportunity offered by these bioderived precursors is the development of the so-called transient electronics. Bioderived precursors can be tailored to tune their biodegradability which allows for the reduction of end-of-life waste electronic materials.^21^ In this sense, research on bioderived LIG contributes to attaining the Sustainable Development Goals by the United Nations, fostering the implementation of SDGs 9 (Industry, innovation, and infrastructure) and 12 (Responsible consumption and production).

The aim of this review is to provide an overview of LIG obtained from bioderived precursors, their peculiarities, and their performance in applications. The reader can gain insight into how the different bioderived precursors, with their unique chemical properties and structure, can result in various forms of LIG, suitable for different applications. The precursors are grouped into three main classes: lignocellulosic materials (wood and leaves and cork), cellulose (paper and nanocellulose), and lignin. Most of them are part of the pulp and paper process or of its byproducts; given the huge volumes of this industry, it is clear why these precursors have attracted many researchers. The relevant properties of the resulting bioderived LIG are described, listed in a table (provided in the Supporting Information), and compared for each class. All of the bioderived precursors are compared with the benchmark of PI-derived LIG and their specific advantages and disadvantages for applications are discussed.

Then the various application scenarios are sorted into three main types of devices: supercapacitors (SC), electrochemical sensors (ES) and physical sensors (PS). The performance of the different bioderived LIGs in each application scenario is evaluated and discussed. Tables summarizing and comparing the performances of the examples reviewed are provided. A critical review is provided, addressing the key advantages and disadvantages of each material class in combination with applications.

## 2 Bioderived Precursors

Several approaches exist for obtaining bioderived carbon, all involving pyrolysis of agrifood^22−27^ or animal wastes.^27,28^ Traditional pyrolytic processes from bioprecursors could be advantageous when large volumes of graphene are needed and no patterning is necessary, as in the case of compounding for the structural reinforcement of polymer materials. On the other hand, laser-induced pyrolysis and LIG benefit from a faster and direct patterning of conductive carbon onto target bioderived precursors/substrates. This is especially beneficial in the case of electronic applications or whenever an integration of graphene-based components into a larger device or system is needed. A hybrid two-step process has also been proposed, with an initial traditional pyrolysis of the precursor to amorphous carbon, followed by a conversion into LIG by laser-induced pyrolysis.^29−35^ This type of LIG is not considered in this review because the focus is on the direct conversion of the bioderived precursors through just laser scribing. Also, other noncarbonaceous substrates for laser patterning have been reported in literature, such as poly(dimethylsiloxane) (PDMS).^36,37^

A detailed description of the photochemical and photophysical conversion mechanisms of bioderived precursors into LIG is provided in a recent review, together with an insight on the role of interfaces in the synthesis.^38^

**Figure 2:**
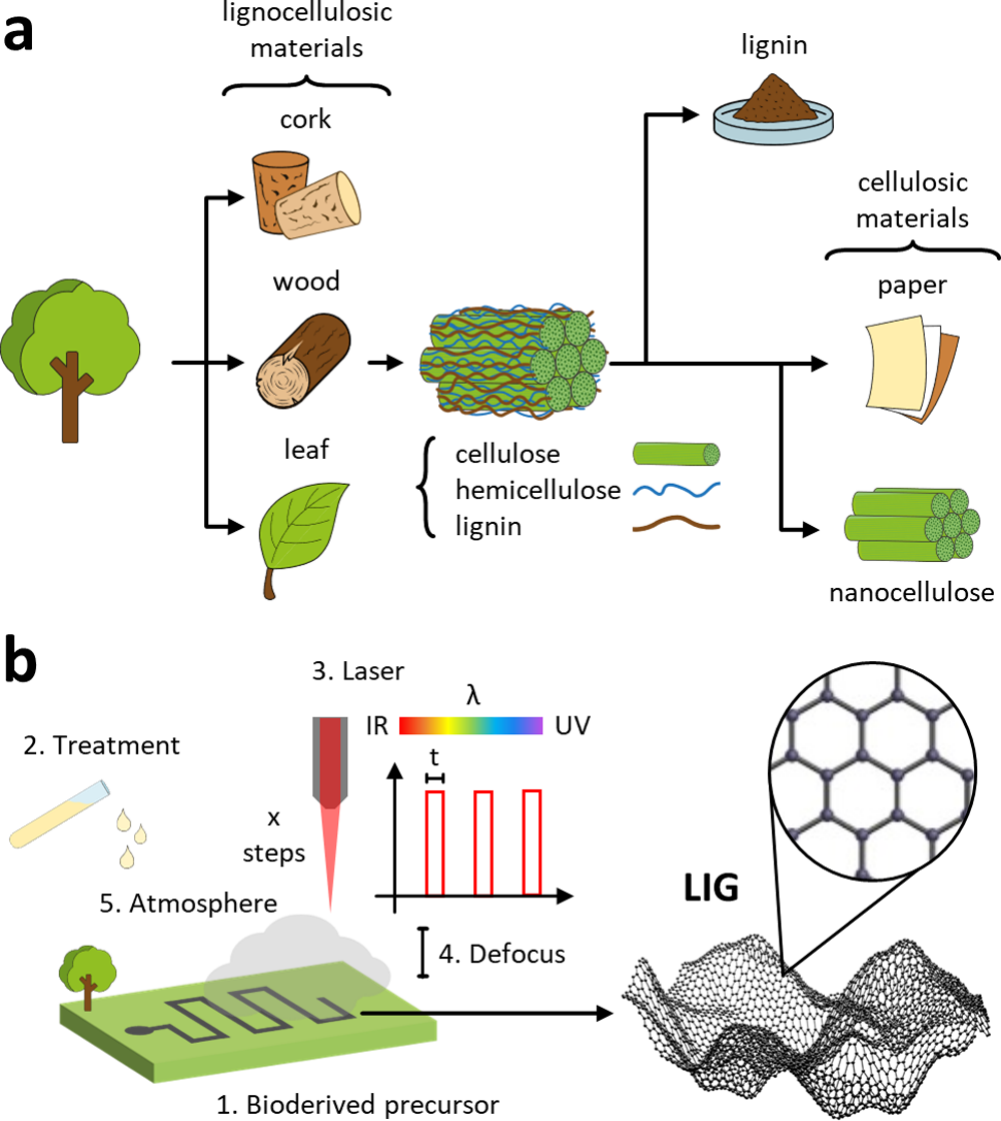
(a) Schematic overview of the main bioderived precursors and their origin/processing. (b) Laser scribing of a bioderived precursor. Variables influencing LIG: (1) chemical composition of bioderived precursors; (2) treatment prior to laser; (3) laser features: wavelength λ, pulse width *t*, number of repeated scribing steps *x*; (4) defocusing of the beam; (5) scribing atmosphere. Graphene network adapted from ref (10). Available under a CC BY 4.0 license.

Almost all bioderived precursors tested so far for LIG production are lignocellulosic materials such as wood or some processed part of it (cork, paper, lignin, and nanocellulose), as highlighted in Figure 2a. The approaches proposed are quite different: in some cases, inhomogeneous raw precursors (wood, cork) or fibrous materials having some processing (e.g., the several types of paper) were tested. In other cases, fractions and byproducts from wood and paper industry (lignin, nanocellulose) with extensive processing and refinement served as precursors, often in the form of sheets/thin films with homogeneous composition. These two groups of precursors have profound differences, especially in possible applications. While the first group of raw, fibrous materials offer obvious advantages in terms of availability and cost, it suffers from inhomogeneity and large structural and composition variability of different sources. Such substrates can hardly meet the requirements for use in devices. Also, it is difficult to integrate these substrates into complete electronic systems. The second group, despite the increased costs related to materials and processing prior to scribing, is prone to a fine-tuning of their properties and preparation in the desired forms (e.g., homogeneous films), with improved chances for device integration.

Many variables can be adjusted to tune LIG properties, hereinafter listed and summarized as 1–5 in Figure 2b.

1. Chemical composition of precursors. Many polymers have been tested, including a wide variety of bioderived precursors. LIG doping with selected heteroatoms is possible by properly choosing the precursors or by the addition of other chemical species prior to scribing.^39−41^
2. Precursor’s treatment prior to lasing. It mostly consists of treatment with fire retardants (FR). This kind of pretreatment is truly relevant for bioderived precursors, typically having poorer thermal and fire stability with respect to high-performance synthetic polymers.
3. Laser features. A first important feature is the type/emission wavelength λ. UV,^4,42−44^ visible.^11,17,45−47^ and IR^4,30,48^ laser sources have been successfully tested, enabling us to choose between photochemical or photothermal routes for the pyrolysis process.^49,50^ Also, the pulse width *t* (continuous, picosecond, and femtosecond)^48,51,52^ of the laser scribing profoundly affects the LIG formation.
4. Fluence H of the laser scribing.^53^ Fluence H is defined as the energy per surface area and it is an important parameter, which also depends on the focusing conditions (4) of the laser and on the number of consecutive multiple laserings on the same spot.
5. Scribing atmosphere. Laser scribing in air, Ar, N_2_, H_2_, or other gas mixtures can lead to very different results in terms of both structure and composition of the obtained LIG. Some precursors can be turned into LIG just and only in an inert atmosphere, while others are prone to LIG even in the air (i.e., in the presence of O_2_).^19,29,38,54,55^

It is worth mentioning a few notes on FR. Despite the fact that the use of FR as additives for pretreatment of bioderived precursors is widespread, an in-depth disclosure of their role in LIG synthesis is not always available, nor is its chemical pathway. Most of the research just adopts it without further investigation of the mechanism. More knowledge exists about the FR themselves, even if they are not specifically involved in LIG synthesis. This is the case of phosphate-based FR, which is the most used in bioderived LIG. When heated, as in the case of the laser scribing, it reacts, turning into polymeric phosphoric acid and creating a char layer. This layer prevents further burning of the material and may contribute to the formation of LIG.^56^

A common set of characterization methods is used to ascertain the creation of a LIG and measure its properties. The main ones are Raman spectroscopy (carbon structure), scanning electron microscopy (SEM) (morphology of the porous structure), and sheet resistance measurements (electrical properties). Other relevant analyses are Brunauer–Emmett–Teller (BET) (surface area analysis), X-ray photoelectron spectroscopy (XPS) and energy-dispersive X-ray spectroscopy (EDX) (elemental composition), X-ray diffraction (XRD) (atomic and molecular structure), and Fourier transform infrared spectroscopy (FTIR) (functional groups). Among them, Raman spectroscopy is the main technique to assess the LIG quality, allowing to identify graphenic/graphitic materials and to differentiate them with respect to amorphous carbons. A complete introduction and overview of Raman spectroscopy of graphene-based materials are provided in.^57^ The Raman spectrum of carbon materials (Figure 1c) is characterized by three main bands (G, D, and 2D), which can slightly differ in width, intensity, or position based on the crystalline structure. The presence of these bands depends on (i) clustering of the sp^2^ phase; (ii) bond disorder; (iii) presence of sp^2^ rings or chains; (iv) the sp^2^/sp^3^ ratio.^58^ The G band (1580 cm^–1^) is connected to the in-plane stretching of C–C bonds, and it gives information about the order of the graphitic structure. The D band (1350 cm^–1^) is due to a breathing mode forbidden in perfect graphene, and it can be observed only in the case of lattice defects. The 2D band (2702 cm^–1^) is derived from the stacking of graphene sheets along the out-of-plane axis and is associated with the properties of multilayer graphene.^57−60^ The intensity ratios of these bands are usually analyzed: a high *I*_D_/*I*_G_ indicates a disordered in-plane structure, while a high *I*_2D_/*I*_G_ suggests a graphenic structure with a piling of multiple graphene sheets.^60−63^

### 2.1 Lignocellulosic Raw Materials

Lignocellulosic are materials that consist of lignin (aromatic-rich), cellulose, and hemicellulose (polysaccharides). Natural sources include trees, their bark, and leaves as well as other plants.

#### Wood and Leaves

Wood is made of cellulose (≈40–45%, fundamental for papermaking), hemicelluloses (≈15–35%), and lignin (≈20–30%, to cement the wood fibers together).^64,65^ Fallen leaves slightly differ in composition, with estimated weight percentages of ≈47% and ≈39% for aliphatic and aromatic compounds, respectively.^66^ These three polymeric components can be seen as the LIG precursors, and they differ in their repetitive units as well as in their structure. The differences in the relative composition of cellulose, hemicellulose, and lignin in the various raw natural materials affect the carbonization pathways and their efficiency, as highlighted in section 2.4.

LIG from wood was first investigated by laser scribing under an inert atmosphere (Ar or H_2_), while scribing in air resulted in just ablation.^19^ The scribing in an inert atmosphere was carried out in a chamber with an IR-transparent ZnSe window purged with the gas and by using a 10.6 μm CO_2_ laser source. An increase in lasering power (and hence fluence) led to structures with smaller pores and higher degrees of crystallinity, as observed by Raman spectroscopy. From TGA analysis, it was shown that the lignin percentages were 26%, 27% and 31% for the samples of pine, birch, and oak, respectively. These results are in agreement with I_D_/I_G_ ratios obtained with Raman spectroscopy, which were 0.85, 0.73, and 0.48 for LIG obtained on the same samples. Indeed, this proved that a higher content of hemicellulose and cellulose, which are more easily decomposed than lignin, results in more defects in the obtained LIG. However, it should be noted that the lignin content of softwoods (pine) normally is higher than for hardwoods (oak, birch): 25–35% against 15–30%.^69^ The sheet resistance of LIG was measured to be as low as ≈10 Ω/□, which is extremely good also compared to that of LIG obtained from synthetic high-performance polymers. The same approach with only inert atmosphere (N_2_ in this case) on pinewood was exploited to assess LIG quality with the variation of the pulse width of a 1064 nm laser.^70^ Going from a pulse width of 10 ns to 10 ps the optimal fluence for LIG decreased from ≈1000 to 660 J cm^–2^, with an increase of sheet resistance from 35 to 179 Ω/□. These results obtained with ns lasers are connected with a less-defective graphene structure, probably because of the lower temperatures reached during pyrolysis.

In order to obtain LIG on wood in ambient air, a pretreatment with chemicals acting as FR is needed. One of the first results with this approach showed excellent results: plywood treated with a phospho-ammonium-boron FR led to a sheet resistance of ≈8 Ω/□.^29^ Recently, an iron-catalyzed laser-induced graphitization on native wood and thin wood veneers has also been reported: the technique is based on a wood coat with an iron-tannic acid ink.^71^ The use of this ink was preferred over commonly used iron salts (chlorides, sulfates, or nitrates) because no hazardous gases are created during laser exposure. Also, tannic acid in the ink is known as a precursor itself for carbonization. Upon laser scribing, native wood samples were damaged and ablated, while the ink-coated ones resulted in a carbonized layer with no cracks. The paint penetrated only around 50 μm of the wood surface. As a result, a 20 μm thick LIG was obtained with a sheet resistance of 20 Ω/□. EDX measurements showed that iron was distributed over the whole surface as core–shell Fe/C nanoparticles. Tensile tests showed that laser treatment and LIG conversion did not reduce the strength of the material. To investigate the catalytic role of iron, samples with an iron-free ink were lasered. These samples needed at least two laser scribing passes to become conductive (60–70 Ω/□) but were composed of amorphous carbon instead of LIG.

**Figure 3:**
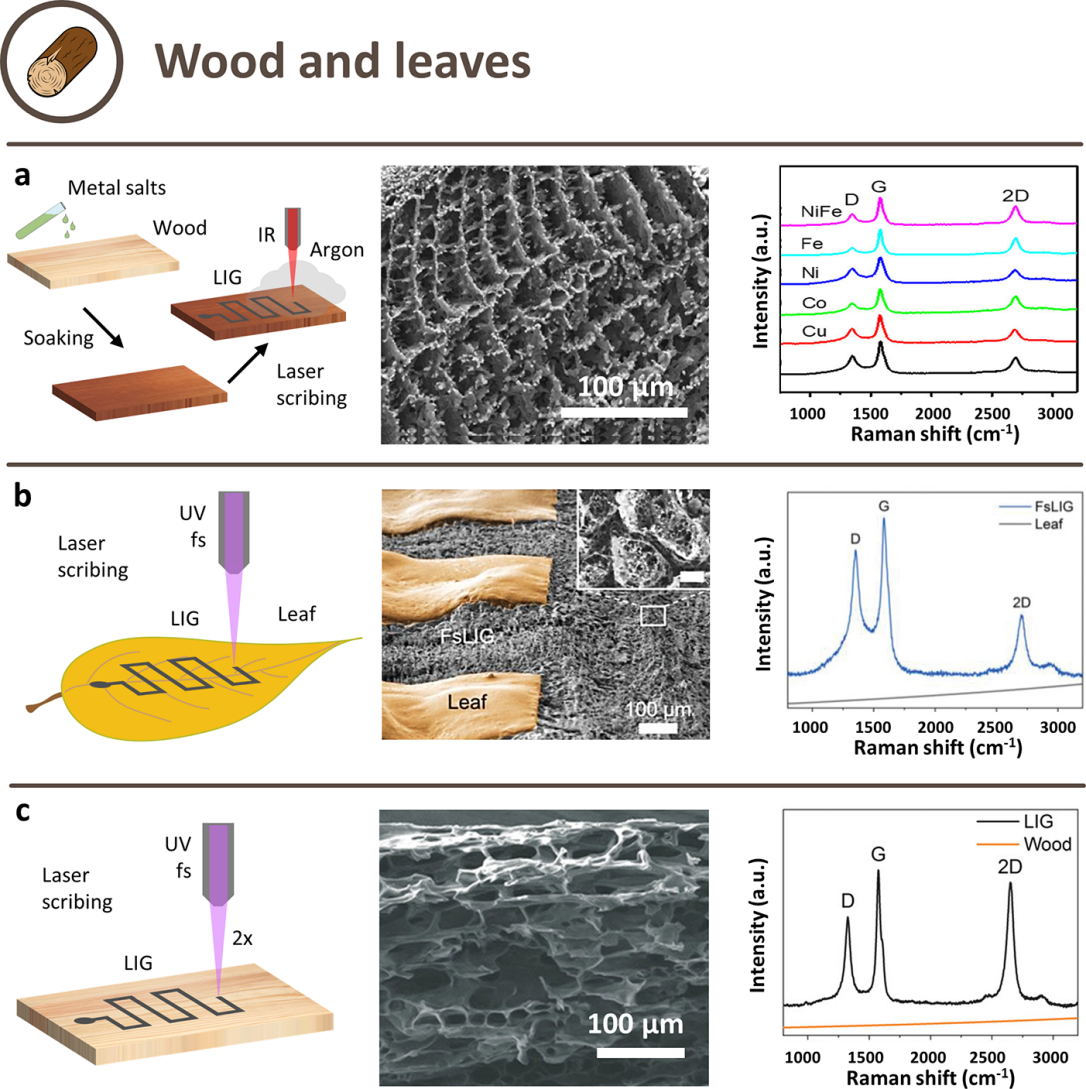
Schematic illustration of possible approaches to obtain LIG from wood-derived precursors and their respective SEM images and Raman spectra. a) Combined approach of precursor treatment with FR and scribing in an inert atmosphere. Reprinted with permission from ref (67). Copyright 2018 American Chemical Society. (b) Approach with a femtosecond laser. Reprinted with permission from ref (66). Copyright 2022 Wiley–VCH. (c) Combined approach of femtosecond laser and multiple scribing. Reprinted with permission from ref (68). Copyright 2019 Wiley–VCH.

A hybrid approach combining inert (Ar) atmosphere and precursor treatment (Figure 3a) was carried out by soaking cedar wood in different metal nitrate solutions (Cu, Co, Ni, Fe, NiFe), and resulted in simultaneous LIG creation and *in situ* formation of metal nanoparticles.^67^ Investigations by XPS showed that the metal salts were converted to elemental metal nanoparticles, which was expected since cellulose is a reducing polysaccharide that is able to convert metal nitrates into metal nanoparticles. Nitrate-soaked wood could be converted into LIG at reduced power, around 60% of the value of untreated wood. Particularly interesting is the case of Fe nitrate: Raman spectra showed typical LIG bands with the lowest I_D_/I_G_ ratio in the series of nitrates (0.3) and the LIG-Fe had a quite low sheet resistance of <7 Ω/□. The obtained NP-decorated LIG had a quite thick (≈80 μm) porous structure.

A different approach was substituting the continuous laser sources with fast pulsed ones (Figure 3b), which dramatically affected LIG scribing from wood. LIG has been obtained by scribing with a UV fs laser under ambient conditions on various lignocellulosic materials: softwoods (pine), hardwoods (rosewood, narra padauk, basswood), engineered woods (pressed wood, plywood), leaves and bamboo.^66,68^ The short pulse width and wavelength made it possible to convert the bioprecursors without burning them and with a very low ablation of the material. So-obtained LIG on wood had a sheet resistance of 10 Ω/□; conductive tracks with a width of 40 μm were fabricated, a significant improvement in resolution with respect to IR laser scribing.^68^ A one-step conversion was possible upon pretreatment with a KMnO_4_ solution, which led to MnO_2_-doped LIG, with similar performances to pure LIG.^68^ Bamboo was also laser scribed by a fs laser (522 nm).^72^ Having a similar lignin content as wood (≈25%) but lower than leaves,^73^ formed good quality LIG with relatively good conductivity, but just after multiple steps of irradiation.^72^

The case of scribing on leaves was particularly interesting and peculiar. Leaves also contain some minerals, such as whewellite (calcium oxalate monohydrate) and sulfur salts (CaS): upon laser-mediated thermal activation, they interact creating nucleation sites for the LIG, as confirmed by XRD and modeled by density functional theory calculations.^66^ Leaves strongly absorbed UV light and mostly reflected near-IR light; thus, they were converted to LIG when exposed to a one-step UV (346 nm) or visible (520 nm) fs laser, but not with a IR laser (1040 nm). The cell structure of the leaves created a very porous LIG with macropores of 20–50 μm in size while retaining a good sheet resistance of around 25 Ω/□.

The same multistep approach (Figure 3c) used in^68^ and^72^ has been demonstrated with a IR laser on various bioderived precursors.^29^ The wood (pine and oak) was first charred with a largely defocused laser and then converted to LIG with a focused one. Also, coconut shells and potato skins were successfully carbonized with the same process. When UV lasers (275–363 nm) were used for the second step, no LIG could be obtained.

#### Cork

Cork is a special lignocellulosic material which is harvested from the bark of cork oaks (Quercus suber).^74^ The core components of cork are suberin (≈45%), lignin (≈20%), and polysaccharides (cellulose and hemicellulose) (≈12%).^74,75^ Cork can be classified into two types: natural cork and agglomerated cork. The latter consists of small pieces of native cork glued together. Depending on the harvesting (virgin tree, first-reproduction, second-reproduction, or successive), natural cork has distinct characteristics in terms of structure, thickness, porosity (and thus density), and strength.

**Figure 4:**
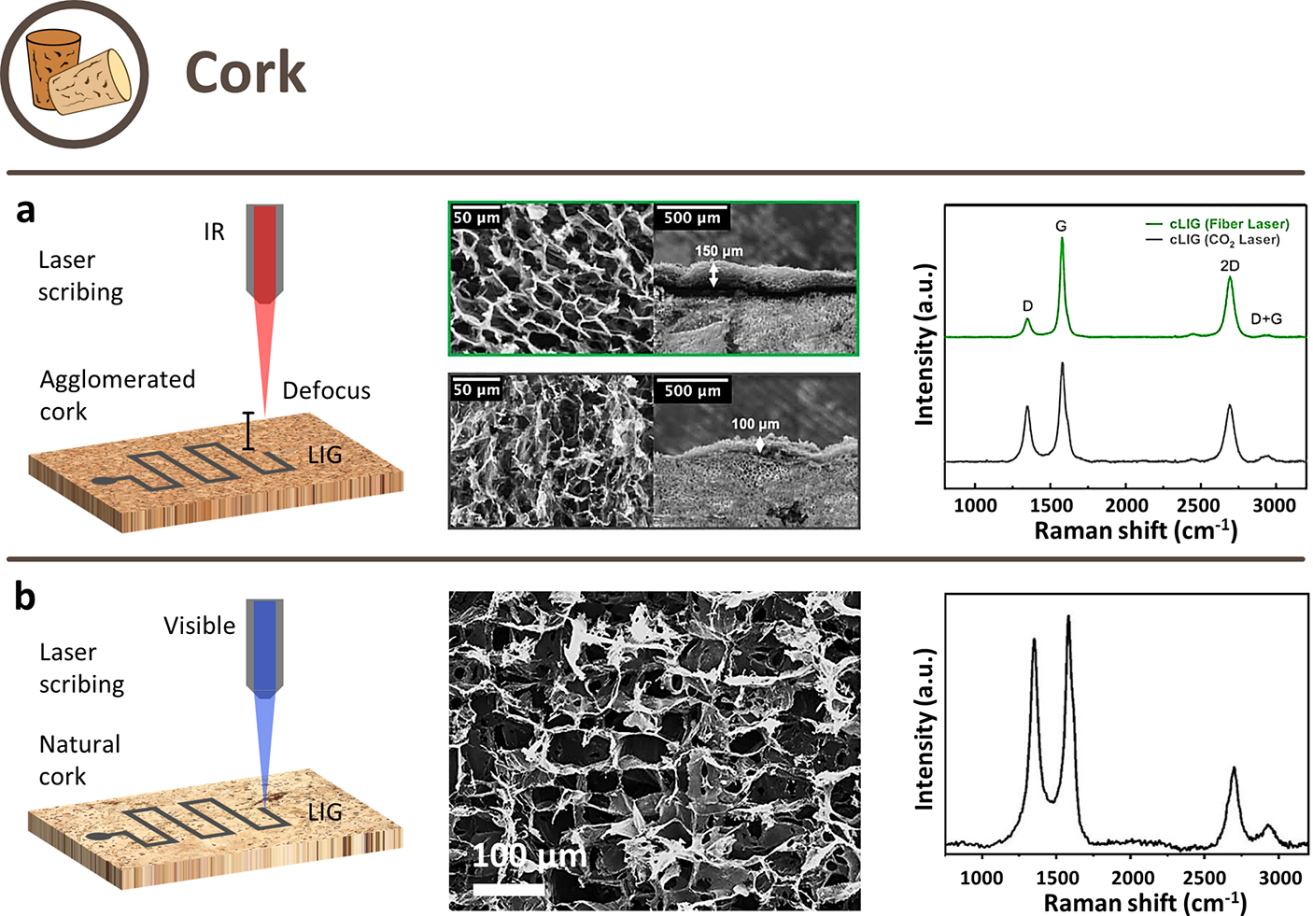
Schematic illustration of possible approaches to obtain LIG from cork-derived precursors and their respective SEM images and Raman spectra. (a) Defocused single-step carbonization in ambient air. Reprinted with permission from ref (76). Available under a CC BY 4.0 license. (b) Single-step carbonization in ambient air. Reprinted with permission from ref (77). Copyright 2022 American Chemical Society.

The first approach for cork-derived LIG was using an IR laser with eventual defocusing during scribing in ambient air, on both natural and agglomerated cork (Figure 4a).

A LIG layer with a thickness of ≈300 μm and sheet resistance of 115 Ω/□ was obtained from native cork with a 10.6 μm IR laser.^78^ The use of a 1.06 μm fiber laser allowed to preserve more of the porous structure and create a larger and more uniform layer of LIG with respect to the 10.6 μm one but, in this case, agglomerated cork was used for the comparison.^76^ A decreased I_D_/I_G_ ratio of 0.2 (vs ≈0.5) in Raman spectra indicated fewer defects, also reflected in the sheet resistance, which was lowered to around 10 Ω/□ (about 1 order of magnitude lower than what is found in literature). Furthermore, it was shown that a slight defocus during scribing can increase the LIG quality.

The second approach consisted of the use of visible or UV lasers (Figure 4b). Both agglomerated and natural cork were converted to LIG by a 355 nm laser source: the LIG layer in this case was just 100 μm thick, three times smaller than with IR laser scribing.^79^ The resulting LIG showed the typical bands in the Raman spectrum and had an *I*_D_/*I*_G_ ratio of ≈0.4 and an *I*_2D_/*I*_G_ ratio of ≈0.37 for both cork types. Quite interestingly, SEM micrographs revealed that the pore structure of cork was mostly conserved, with the cell walls converted into thin layers of LIG. The sheet resistance of agglomerated cork was lower than the one of natural cork (75 Ω/□ against ≈90 Ω/□), and it was highlighted that natural cork showed anisotropic resistance in the two planar directions. Similar results were obtained on disks of natural cork stoppers by scribing with a 450 nm laser in an ambient atmosphere.^77^ BET analysis showed that the porosity of cork increased after converting it into LIG (specific surface area from 1.8 m^2^/g to 4.6 m^2^/g and pore volume from 0.00774 cm^3^/g to 0.02281 cm^3^/g), which make cork-derived LIG excellent for electrochemical applications due to the enhanced charge transfer rate in the electrodes and good energy storage. Furthermore, contact angle measurements showed a decrease from ≈103° to ≈81°, which results in a larger contact area when wet. Moreover, cork could be pretreated with H_3_BO_3_ to increase the electrical performances (sheet resistance from 46 Ω/□ to 38 Ω/□), which resulted in LIG decorated with boron microcrystals.^77^

### 2.2 Cellulosic Materials

Cellulose is isolated in the pulp and paper production process, which can be divided into two parts. The first one, pulping, consists of the conversion of the starting lignocellulosic material into pulp by removing as much lignin as possible from the precursor. The second part is the group of steps to reach the finished product, namely paper. The starting material for the whole process is often wood, but even rags, flax, cotton linters, and bagasse (a sugar cane residue) can be used.^65^ Depending on the specific applications, many additives can be added to paper products (e.g., inorganic fillers such as CaCO_3_),^80^ and may influence LIG formation and performance.

#### Paper

Paper was one of the first bioderived materials which were converted into graphene by laser-induced pyrolysis.^20^ The use of paper as a substrate for devices is a good solution when a trade-off between cost and performance is required: not only paper is a cheap, lightweight, flexible, generally hydrophilic, and with a high loading capacity material (important in many applications), but it is also recyclable and biodegradable, which are relevant features for sustainability.^81^ Moreover, paper is easily available and exists in different types, sizes, thicknesses, and finishes, which can be tuned according to the application.^80^ An analysis of different types of paper used to obtain LIG for various applications is carried out in this section. It is worth mentioning phenolic paper,^82^ a tough, low-cost, and nonconductive common substrate for the fabrication of printed circuit boards. However, it is not included in the review since it is only a partially bioderived precursor (synthetic phenolic resin is one of the main components).

**Figure 5:**
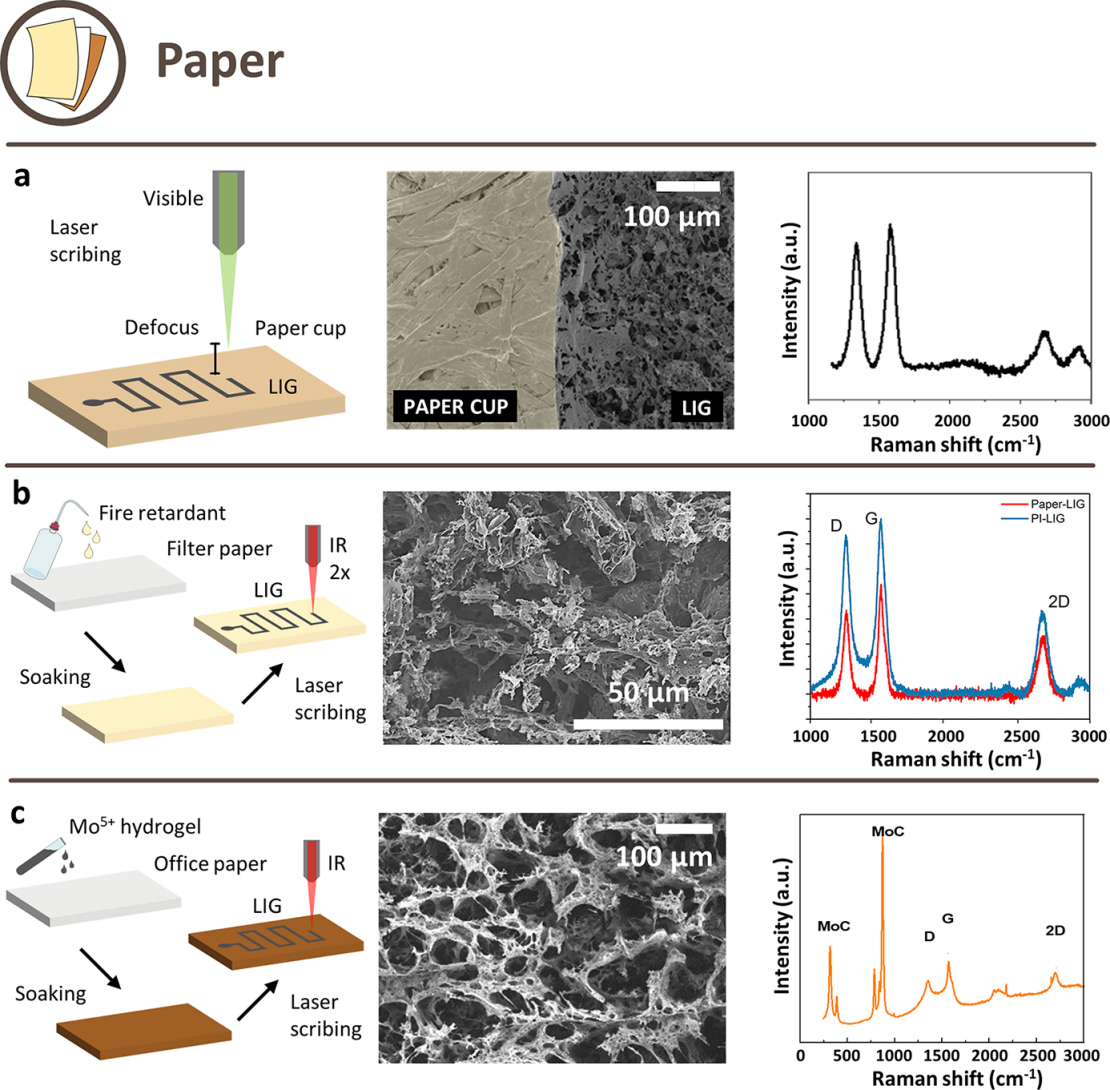
Schematic illustration of possible approaches to obtain LIG from paper-derived precursors and their respective SEM images and Raman spectra. (a) Approach with single-step defocused scribing. Reprinted with permission from ref (83). Copyright 2022 Elsevier. (b) Approach with FR pretreatment and multistep scribing. Reprinted with permission from ref (84). Copyright 2020 Elsevier. (c) Approach for MCG paper. Reprinted with permission from ref (85). Copyright 2020 Elsevier.

The first approach for paper-derived LIG production consisted of defocused single-step scribing (Figure 5a). Different paper types (filter, kraft, sulfite, and paperboard), were scribed with a 10.6 μm CO_2_ laser under ambient atmosphere, with a large defocus (4–12 mm).^20^ A 665 g/m^2^ paperboard was proven to be an excellent precursor for LIG: the conductive track had a thickness of around 320 μm and a porous structure with an average pore size of around 100 nm and was decorated with aluminosilicate nanoparticles, coming from commonly used paper additives. With optimized parameters, a sheet resistance of ≈14 Ω/□ could be achieved. The same paperboard was studied in similar conditions, and the obtained sheet resistance was ≈11 Ω/□.^86^ In another study, it was scribed in unknown (likely defocused) conditions but showed worse *I*_D_/*I*_G_ ratio in its Raman peaks (1.21 against 0.71).^61^ This approach has also been applied to cardboard.^87^ Scribing with a 532 nm continuous wave Nd:YAG galvo laser has been tested on paper cup (similar to paperboard), inner surface of milk cartons, and colored origami paper (similar to office paper), with a range of defocus distances.^83^ The best result for office paper was 105 Ω/□, a value that is slightly higher than other works. Office paper was also the subject of a peculiar study: a commercial pencil was used to treat the precursors prior to scribing it with a 10.6 μm CO_2_ laser in defocused conditions.^88^

A different approach was the pretreatment with FR to allow for multistep carbonization in ambient air with a 10.6 μm CO_2_ laser. Usually, the first step was operated with a more defocused beam, and the second step with less defocusing or in focus (Figure 5b). In some cases, also single-step carbonization was reported. Cotton paper was soaked with various FR: a commercial phosphate-based one, FeCl_3_ and boric acid. The latter showed the best result in terms of the quality of produced LIG, having a sheet resistance of ≈40 Ω/□.^89^ However, the best sheet resistance value obtained for LIG from paper had been <5 Ω/□.^29^ An 800 g/m^2^ paperboard, with kaolin and CaCO_3_ as additives, was instead treated with a solution of sodium borate.^90^ Also filter paper could be converted after being treated with an aqueous solution of ammonium sulphamate, urea and other components,^91^ phosphate-based,^92,93^ sodium borate-based^94^ FR, or a commercial one,^62^ obtaining similar results in terms of sheet resistance (around 30–60 Ω/□). An interesting application has been proposed for this approach: triboelectric nanogenerators were created by combining multistep pretreated paper-derived LIG and PI-derived LIG, showing good performance.^84^ In a further study,^95^ two-step lasering of activated charcoal filter paper and single-step of regular filter paper were analyzed, both pretreated with a phosphate-based FR (for the charcoal filter it was applied between the two steps). It was proven that the presence of activated charcoal did not provide a substantial advantage for the process of formation of LIG from paper. The best parameter combinations showed ≈180 and ≈100 Ω/□, respectively, for activated carbon and regular filter paper. The second precursor was also single-step scribed with a pulsed 355 nm UV laser, resulting in a slightly higher sheet resistance value of 125 Ω/□.^96^

Recently, a new technique specific to ES has been proposed.^97^ Filter and office paper were soaked in a sodium tetraborate water solution (FR) and subsequently dried. Then, paper sheets were wax printed with the purpose of inducing hydrophobicity through their volume, for impermeabilization of the substrate and further use in ES production. LIG could then be obtained through single-step irradiation with a 10.6 μm CO_2_ laser operating in air and resulted in a sheet resistance as low as ≈60 Ω/□ for filter paper and ≈220 Ω/□ for office paper. Additives used in office paper fabrication could be clearly identified even after lasering, mainly consisting of large salt agglomerates of CaCO_3_.

The third approach for paper-derived LIG consists of a specific class of pretreated paper substrates/precursors: molybdenum carbide-graphene (MCG) paper (Figure 5c). This term groups different kinds of paper sprayed with a metallic-hydrogel (Mo^5+^-gelatin, in an aqueous solution) and then converted to porous and conductive MCG composites by laser scribing. Six MCG coming from commercial paper substrates were scribed with a 10.6 μm CO_2_ laser under ambient environment, to be used as paper-based 3D foldable devices.^98^ The obtained LIG/MCG had a good electrical resistance of 30 Ω/□, and it showed a resilience to repeated folding operations. Several types of water-soluble polymers, including gelatin, poly(ethylene oxide) (PEO), and poly(vinylpyrrolidone) (PVP) were mixed with the Mo ions for tests, and gelatin-based ink led to a much lower sheet resistance. Similar results on MCG/LIG were obtained on fiber paper by various groups.^85,99^

An isolated example was cardboard scribed with a 532 nm continuous wave laser with no pretreatment nor defocusing or multiple lasing, and indeed the result was not as good as the others reported in the literature.^100^

2.2.2

Nanocellulose is an emerging bionanomaterial that comes in two primary forms: cellulose nanocrystals (CNC) and cellulose nanofibers (CNF).^101^ CNC are rod-like nanoobjects made of purely crystalline cellulose, with a diameter of 3–10 nm and aspect ratio between 5 and 50, sometimes higher.^102^ Instead, CNF present both crystalline and amorphous regions, with a diameter of 5–30 nm and an aspect ratio usually >50.^102^ CNC and CNF exhibit the intrinsic properties of cellulose (i.e., low density, renewability, nontoxicity, biocompatibility, biodegradability), plus the specific properties associated with the nanoscale.^103−105^ These properties are exploited in many different industrial applications, mostly in the medical field (tissue engineering, drug delivery),^104^ advanced materials science (lightweight polymer nanocomposites and optically active materials),^103,106,107^ electronics,^108,109^ and food packaging.^103,110,111^

One of the main drawbacks of nanocellulose is the strong interaction with water (large swelling and quick gel formation^112^), which compromises the oxygen transmission rate, a parameter that can have an impact on the degree of graphitization when LIG scribing is considered.^38^

Nanocelluloses are commercially available in different grades and with different surface functionalizations,^113,114^ which may affect the performance and conditions needed for LIG. However, upscaling of processing is challenging and requires many expensive and demanding steps, resulting in a bigger environmental impact.^103^

**Figure 6:**
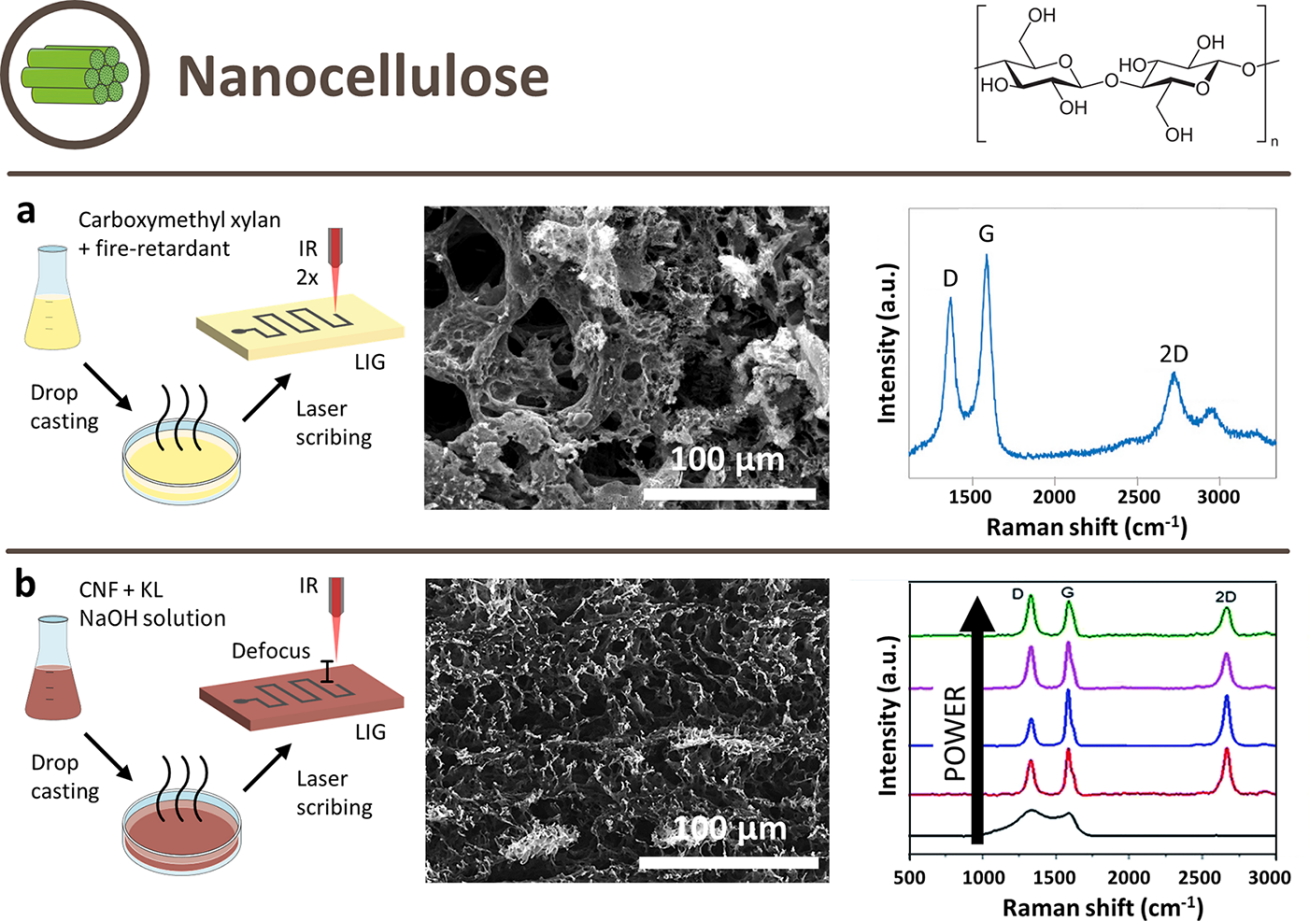
Schematic illustration of possible approaches to obtain LIG from nanocellulose-derived precursors and their respective SEM images and Raman spectra. (a) Approach with FR and multistep scribing. Reprinted with permission from ref (95). Copyright 2022 Elsevier. (b) Approach with defocused scribing. Reprinted with permission from ref (115). Available under a CC BY-NC 3.0 license.

The approaches for LIG from nanocellulose all include different combinations of multistep lasering (Figure 6b), pretreatment with FR (Figure 6a), beam defocusing (Figure 6b), and inert atmosphere. In agreement with the results obtained from the other cellulosic material (i.e., paper), FR itself should be enough to allow for LIG formation, but in the literature, a defocus or multiple lasering is always used in addition.

CNC was first obtained from pineapple leaf fiber, a rich source of cellulose (≈60 wt %) that can be easily delignified. Pineapple-derived CNC were chosen because of their aspect ratio, which the authors hoped would positively influence the LIG structure.^116^ The CNC were functionalized with commercial FR (no information on the composition), compacted into tablets, and then single-step lasered with a 10.6 μm CO_2_ laser under a N_2_ atmosphere to obtain LIG nanopowder. The lasered CNC powder was mixed with sodium carboxymethyl cellulose (CMC) binder to create an ink, which was applied on a tracing paper substrate using hand-drawing techniques. Subsequent scribing with a single-step lasering process resulted in ≈10 μm thick LIG electrodes. The best configuration was CNC tablets, which showed a sheet resistance value of ≈600 Ω/□.

Porous CNF papers were obtained from a CNF solution by vacuum freezing and pressing, and then multistep scribed in air with a IR CO_2_ laser.^117^ CNF films were successfully carbonized with a single-step process with both 10.6 μm^117^ and a 1045 nm fs laser coupled with a large defocus.^118^ The latter produced LIG with a sheet resistance value comparable to the PI-derived one. The reason why in the first case LIG was obtained without defocusing was attributed to the exceptionally low oxygen permeability and the presence of hydroxyl groups and sodium in CNF. More hydroxyl groups mean greater amounts of moisture, which evaporate to suppress temperature increase during heating. On the other hand, sodium lowers the activation energy of the dehydration step and helps the growth of carbonaceous materials that can be transformed into graphitic carbon at higher temperatures (also, a control experiment using a CNF in which sodium was replaced with hydrogen demonstrated that only amorphous carbon was produced by laser exposure).^117^ Interestingly, films composed of CNF and kraft lignin (KL) in a 1:5 mass ratio were converted into LIG with a 10.6 μm CO_2_ laser in ambient conditions, without defocus.^115^ An interesting study investigated spin-coated nail polish films, whose main components are nitrocellulose, a mixture of nitric esters of cellulose (the main carbon source for LIG), organic montmorillonite (an efficient FR) and a dye (may promote the formation of LIG by enhancing light absorption).^119^ The films were successfully scribed with a 405 nm laser in an ambient atmosphere in a single step. This may open the path for different varnish applications, but the sustainability and toxicology of nail polishes should be taken into account.

Also, hemicelluloses were used as LIG precursors: solvent-cast carboxymethyl xylan films treated with phosphate-based FR were multistep scribed with a 10.6 μm CO_2_ laser in ambient condition, with resulting performances close to cellulosic films.^95^

### 2.3 Lignin

Lignin is the most abundant naturally occurring aromatic polymer, mainly constituted of three different monolignols in a complex cross-linked structure, p-coumaryl, coniferyl, and sinapyl alcohols, also called, respectively, H, G, and S lignin. Different bioderived precursors contain lignin with different concentrations of the three monolignols, which results in different chemical and physical properties.^80,120^ Interested readers can deepen their knowledge with refs (121) and (122).

Lignin is a low-value waste product of the paper industry (e.g., pulping mills and biorefineries), extracted in massive quantities from cooking liquor during the pulping process. Depending on the chemicals adopted as liquors, two main groups of processes can be discerned, and thus, different qualities and properties of the products: alkaline pulping (KL and soda lignins) and sulfite pulping (lignosulfonates (LS)). A minor branch is organic solvent pulping (organosolv lignins), which is a more experimental process.^123^ Compared to polysaccharides, lignins are characterized by a high C/O ratio and high thermal stability,^80^ features which makes lignin an excellent candidate precursor for LIG. Another good advantage of lignin is that it can be processed to obtain films, sheets, etc., thus easily be adapted to different application fields.^80,124,125^ However, it is important to remark that, in order to produce films, lignin is often mixed with synthetic polymers to compensate for its bad mechanical properties, mainly brittleness; thus, the goal of replacing synthetic precursors is not fully achieved.

**Figure 7:**
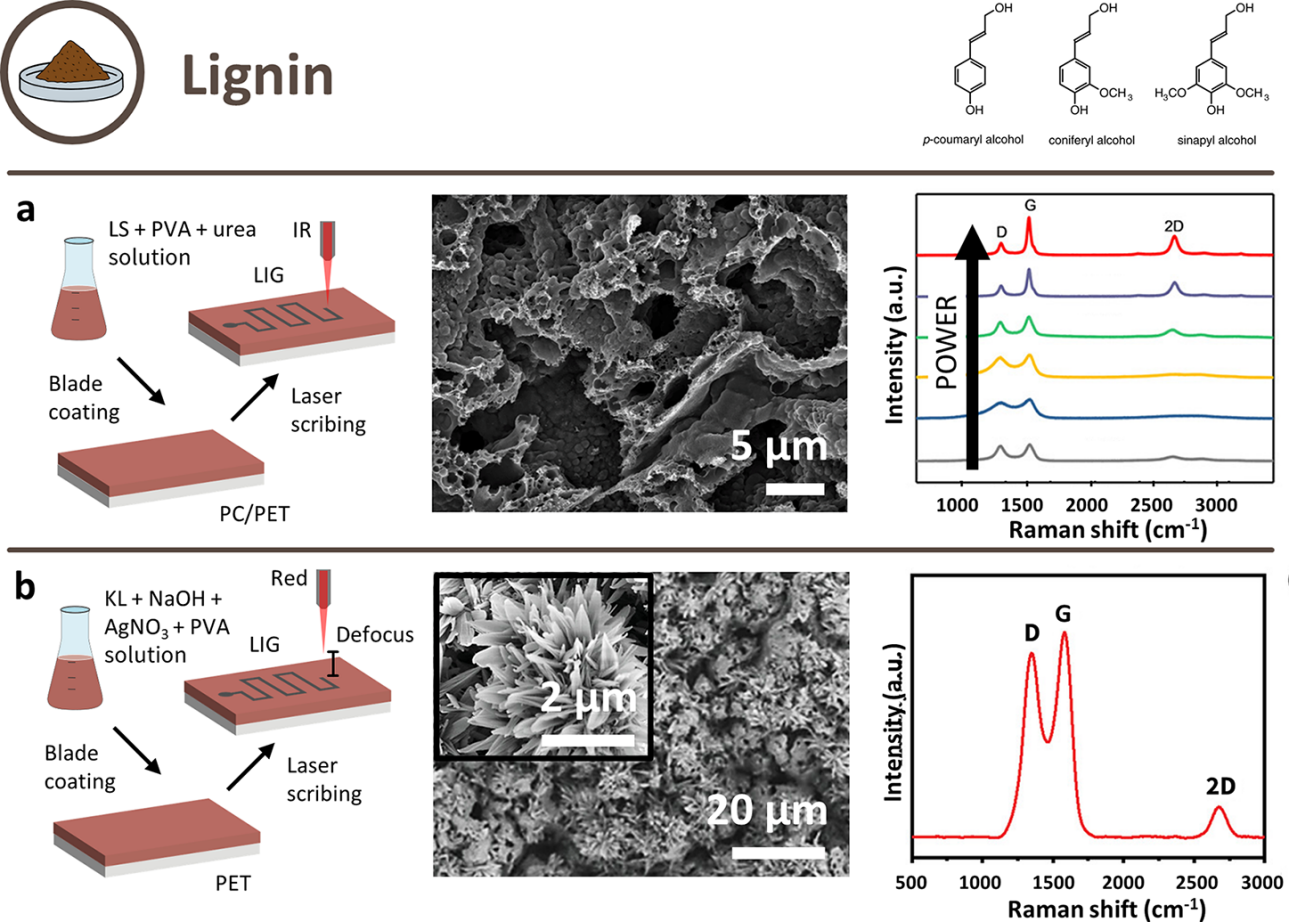
Schematic illustration of possible approaches to obtain LIG from lignin-derived precursors and their respective SEM images and Raman spectra. Chemical structures of monolignols reprinted with permission from ref (126). Copyright 2001 American Society of Plant Biologists. (a) Approach with lignin in PVA matrix. Reprinted with permission from ref (127). Copyright 2019 American Chemical Society. (b) Approach with doping prior to scribing. Reprinted with permission from ref (128). Copyright 2022 Wiley–VCH.

One approach (Figure 7a) for obtaining LIG from lignin was to manufacture films of lignin (both KL and LS) and poly(vinyl alcohol) (PVA). They were single-step scribed with a 10.6 μm CO_2_ laser source in an ambient atmosphere, in one case in defocus conditions (Figure 7b).^125^ They showed excellent results, both in sheet resistance measurements (2.8–4.5 Ω/□) and in Raman peak ratios (*I*_D_/*I*_G_ between 0.33 and 0.39 and *I*_2D_/*I*_G_ between 0.5 and 0.77).^124,125,127,129^ An interesting technique was to use a water lift-off process to remove the nonexposed parts of the films.^124^ This approach^124^ was improved by removing the need of dissolving the unused precursor: a forest-based ink for flexible and printed electronics has been obtained by combining LS and cellulose, with the addition of boric acid as FR, and screen-printed on polyethylene terephthalate (PET) sheets.^130^ The ink pattern was then carbonized with a 10.6 μm CO_2_ laser in ambient conditions and resulted in an excellent sheet resistance value (≈4 Ω/□).Other polymers adopted to obtain lignin films are PEO,^131,132^ polyethersulfone (PES),^133^ and poly(acrylonitrile) (PAN).^134^ Even if the only difference in scribing conditions with respect to PVA was, in one case, the use of a fs laser,^134^ the obtained LIG showed slightly worse performances. Furthermore, it must be said that PES is itself a precursor for LIG.^135^

LIG doping is another approach: in one case. lignin was mixed with carboxymethyl chitosan and spread on wood to obtain O/N/S codoped LIG.^136^ Despite the functional application, not enough characterization was provided to ensure LIG formation and, moreover, it was not evident which material was the actual precursor for LIG. Another example is Ag-doped LIG, obtained by manufacturing a film of lignin with AgNO_3_, KOH, PVA, and scribing it with a 800 nm fs laser in defocused conditions.^128^

Flexible composites were instead obtained by embedding lignin in PDMS,^43,132,137^ and then carbonizing them in ambient air using lasers with different wavelengths (IR,^132^ visible,^137^ UV^43^). In one case, lignin was first dissolved in PEO and then mixed with the elastomer prepolymer mixture.^132^ Even if achieving flexibility and stretchability is extremely interesting from an application point of view (e.g., biomedical field), the LIG characteristics were not as good as in the other approaches, not even with defocused scribing.^43,132^ Another substrate adopted to obtain flexible composites was carbon cloth, which was covered with melted lignin and scribed with a 10.6 μm laser.^138^ No sheet resistance measurements are provided, but the Raman peak ratios are significantly better than those for PDMS composites.

It is also worth mentioning the use of nanolignin as the LIG precursor. NanoKL/CNF films^139^ and nanoKL tablets^140^ were converted into nanodiamonds through laser-induced pyrolysis in an air environment with a 1030 nm femtosecond laser. SEM images showed that the tablets created bubbles when scribed with the laser, but the electrical performances were better than those of films (sheet resistance of 306 Ω/□ instead of 510 Ω/□). Unfortunately, the only example of pure lignin films (drop-casted aqueous solution), which would have had a great advantage in terms of sustainability (less processed than nanolignin and no chemicals added) resulted in just amorphous carbon, as confirmed by Raman and poor conductivity.^141^

### 2.4 Comparison among Bioderived Precursors

**Figure 8:**
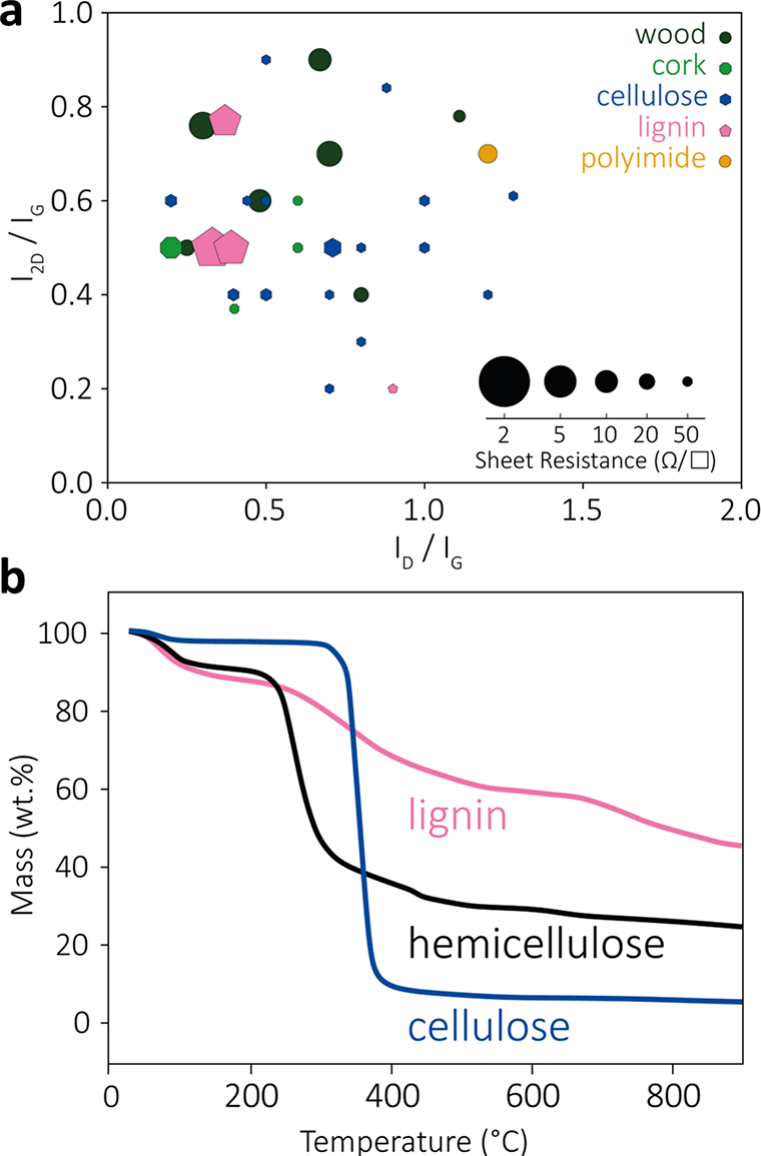
(a) Overview of different precursors showing *I*_2D_/*I*_G_ over *I*_D_/*I*_G_ and their relative sheet resistance (mentioned references with not complete results are not shown). Sheet resistance values of ≥50 Ω/□ have been represented with the smallest symbol dimension. (b) Thermogravimetric analysis of cellulose, hemicellulose, and lignin. Reprinted with permission from ref (143). Copyright 2007 Elsevier.]

To compare and summarize the properties of LIG from the different bioderived precursors, three characteristic properties have been selected and shown in Figure 8a: the band ratios *I*_D_/*I*_G_ and *I*_2D_/*I*_G_ from Raman spectra (shown on the *x* and *y* axes, respectively) and the sheet resistance of the obtained LIG materials (proportional to the diameter of the circles for each point). Data shown in the plot are taken from the publications reviewed in Tables 1–5. Different colors are assigned to the different subclasses of precursors discussed so far: wood, cork, paper, nanocellulose, and lignin. A single reference point is also given for synthetic PI, taken from,^1^ to provide a mean of comparison. The best quality of the crystalline graphitic structure in LIG is associated with low *I*_D_/*I*_G_ and high *I*_2D_/*I*_G_ (points in the upper left corner of the graph). This combination should result in improved conductivity (i.e., lower sheet resistance) and, although there is some variation, there is indeed a general trend toward lower sheet resistivity. As reported in Tables 1-5, most precursors were scribed with a 10.6 μm infrared CO_2_ laser, because the instrument is cheap and easy to use. The choice of laser source did not have a clear trend on the resulting LIG properties, with few exceptions. A first notable exception is the structure of cork, which could be preserved by using a lower wavelength (1.06 μm instead of 10.6 μm).^76^ A second is the influence of smaller wavelengths and/or pulse widths, which allows to convert some precursors without any pretreatment while also providing unique properties.^66^ Almost all bioderived precursors were laser scribed in an ambient atmosphere, but in very few cases, wood and nanocellulose were scribed in an inert atmosphere (Ar, N_2_, H_2_).

**Table 1:** Summary of All Lignocellulosic Precursors (Excluding Cork) Showing Their Raman Ratios, Electrical Properties, And Application^a^

ref	Precursor	Precursor treatment	λ	Laser settings	Atmosphere	*I*_**D**_/*I*_G_	*I*_2D_/*I*_G_	*R*_s_ (Ω/□)	Application
(19)	Wood	No	10.6 μm	No	Ar, H_2_	0.48	≈0.6^b^	≈10	SC
(70)	Wood	No	1064 nm	No	N_2_	1.11	0.78	35	n.a.
(29)	Wood	FR (pentaerythritol tetraphosphate ammonium salt)	10.6 μm	No	Air	≈0.7^b^	≈0.7^b^	≈8	n.a.
(29)	Wood	No	10.6 μm	Multistep and defocus	Air	≈0.8^b^	≈0.4^b^	<5	n.a.
(29)	Coconut shell Potato skin	No	10.6 μm	Multistep	Air	≈0.8^b^	≈0.7^b^	<5	SC
(71)	Wood	Tannic acid	10.6 μm	No	Air	0.25–0.8	0.5–0.74	20	PS, touch electronics, electroluminescent
(67)	Wood	Metal salts	10.6 μm	No	Ar	0.3	0.76	<7	oxygen evolution reactions
(68)	Wood	No	343 nm	No	Air	0.67	≈0.9^b^	10	PS
(68)	Wood	KMnO_4_	343 nm	No	Air	≈1.3^b^	≈0.5^b^	10	SC
(68)	Leaves	No	343 nm	No	Air	≈1.5^b^	≈0.6^b^	n.a.	PS
(66)	Leaves	No	346 nm 520 nm 10.4 μm	No	Air	≈0.8^b^	≈0.4^b^	23.3	SC
(72)	Bamboo	No	522 nm	No	Air	0.7	≈0.5^b^	n.a.	SC
(1)	Polyimide	No	10.6 μm	No	Air	1.2	0.7	15	SC

a Acronyms: FR = fire-retardant, SC = supercapacitor, PS = physical sensor, n.a. = no value available.

b Extracted from figures because no values were given.

**Table 2:** Summary of All Cork Precursors Showing Their Raman Ratios, Electrical Properties, and Application^a^

ref	Precursor	Precursor treatment	λ	Laser settings	Atmosphere	*I*_D_/*I*_G_	*I*_2D_/*I*_G_	*R*_s_ (Ω/□)	Application
(78)	Cork	No	10.6 μm	No	Air	≈0.6^b^	≈0.6^b^	115	Triboelectric nanogenerator
(76)	Agglomerated cork	No	1.06 μm 10.6 μm	Defocus	Air	0.2	0.5	9.86	SC
(79)	Agglomerated cork Natural cork	No	355 nm	No	Air	≈0.4^b^	≈0.37	75	PS
(77)	Natural cork	No	450 nm	No	Air	0.6	0.5	46	SC
(1)	Polyimide	No	10.6 μm	No	Air	1.2	0.7	15	SC

a Acronyms: SC = supercapacitor, PS = physical sensor, n.a. = no value available.

b Extracted from figures because no values were given.

**Table 3:** Summary of All Paper Precursors Showing Their Raman Ratios, Electrical Properties, and Application^a^

ref	Precursor	Precursor treatment	λ	Laser settings	Atmosphere	*I*_D_/*I*_G_	*I*_2D_/*I*_G_	*R*_s_ (Ω/□)	Application
(20)	Paperboard	No	10.6 μm	Defocus	Air	0.71	≈0.5^b^	≈14↑	ES
(86)	Paperboard	No	10.6 μm	Defocus	n.a.↑	n.a.	n.a.	11	ES
(61)	Paperboard	No	10.6 μm	Supposed defocus	Air	1.21	≈0.4^b^	n.a.	-
(87)	Cardboard	No	10.6 μm	Defocus	n.a.↑	n.a.	n.a.	n.a.	ES
(83)	Colored paper	No	532 nm	Defocus	n.a.↑	≈0.8^b^	≈0.3^b^	105	ES
(88)	Office paper	Pencil layer	10.6 μm	Defocus	Air	0.24	0.51	n.a.	ES
(29)	Cotton fabric	FR (pentaerythritol tetraphosphate ammonium salt)	10.6 μm	Multistep	Air	≈0.6^b^	n.a.	<5	-
(89)	Cotton paper	FR (pentaerythritol tetraphosphate ammonium salt)	10.6 μm	Supposed multistep	Air	≈1.0^b^	≈0.6^b^	≈40	-
(90)	Paperboard	FR (sodium borate)	10.6 μm	Multistep	n.a.↑	≈0.9^b^	n.a.	n.a.	PS
(92)	Filter paper	FR (ammoniumorthophosphate)	10.6 μm	Multistep	n.a.↑	0.5	≈0.4^b^	32	PS
(93)	Filter paper	FR (ammoniumorthophosphate)	10.6 μm	Multistep	n.a.↑	≈1	≈0.5^b^	40	ES
(94)	Filter paper	FR (sodium borate)	10.6 μm	No	N_2_	≈0.4^b^	≈0.4^b^	30	SC
(62)	Filter paper	FR (potassium borate 1–5%)	10.6 μm	Multistep	Air	0.88	0.53	n.a.	ES
(84)	Filter paper	FR (potassium borate 1–5%)	10.6 μm	No	Air	0.79	0.55	n.a.	Triboelectric nanogenerator
(95)	Filter paper	FR (ammoniumorthophosphate)	10.6 μm	No	n.a.↑	≈0.8^b^	0.5	≈100^b^	PS
(96)	Filter paper	FR (ammoniumorthophosphate)	355 nm	No	n.a.↑	≈0.7^b^	≈0.2^b^	125	PS
(97)	Filter paper Office paper	FR (sodium borate) + wax	10.6 μm	No	n.a.↑	1.28	0.61	56.0	ES
(91)	Filter paper	FR (ammonium sulphamate, urea, water)	10.6 μm	Multistep	Air	0.88	0.84	61.5	circuits
(98)	Office paper MCG	Mo^5+^ hydrogel	10.6 μm	No	Air	≈0.5^b^	≈0.9^b^	60	SC + ES
(98)	Filter paper MCG	Mo^5+^ hydrogel	10.6 μm	No	Air	n.a.	n.a.	45	SC + ES
(99)	Filter paper MCG	Mo^5+^ hydrogel	10.6 μm	No	n.a.↑	n.a.	n.a.	<50	EMF Shielding
(85)	Office paper MCG	Mo^5+^ hydrogel	10.6 μm	No	n.a.↑	≈0.7^b^	≈0.4^b^	≈1000^b^	PS
(100)	Cardboard	No	532 nm	Defocus	Air	≈0.9^b^	n.a.	n.a.	PS
(1)	Polyimide	No	10.6 μm	No	Air	1.2	0.7	15	SC

a Acronyms: SC = supercapacitor, ES = electrochemical sensor, PS = physical sensor, MCG = molybdenum carbide-graphene, FR = fire-retardant, EMF = electromagnetic field, ↑ we assume that when no information is given the process was executed in air, n.a. = no value available.

b Extracted from figures because no values were given.

**Table 4:** Summary of All Nanocellulose Precursors Showing Their Raman Ratios, Electrical Properties, and Application^a^

ref	Precursor	Precursor treatment	λ	Laser settings	Atmosphere	*I*_D_/*I*_G_	*I*_2D_/*I*_G_	*R*_s_ (Ω/□)	Application
(116)	CNC	FR (amino salt, water)	10.6 μm	Defocus	N2	≈1.2^b^	≈0.4^b^	≈600	PS
(117)	CNF	No	10.6 μm	Multistep (CNF paper)	Air	≈1.5^b^	≈0.2^b^	≈2000↑	n.a.
(118)	CNF (glass slide sandwich)	No	1045 nm	Defocus	Air	0.2	≈0.6^b^	≈30↑	n.a.
(119)	Nail polish (nitrocellulose)	FR (montmorillonite)	405 nm	Defocus	Air	0.44	≈0.6^b^	≈100^b^	ES
(115)	CNF and KL	No	10.6 μm	Defocus	Air	≈0.5^b^	≈0.6^b^	≈200	ES
(95)	Carboxymethyl Xylan (hemicellulose)	FR (ammoniumorthophosphate)	10.6 μm	Multistep	Air	≈0.8^b^	≈0.5^b^	186	PS
(1)	Polyimide	No	10.6 μm	No	Air	1.2	0.7	15	SC

a Acronyms: CNC = cellulose nanocrystals, CNF = cellulose nanofibers, KL = kraft lignin, FR = fire-retardant, ES = electrochemical sensor, PS = physical sensor, n.a. = no value available.

b Extracted from figures because no values were given.

**Table 5:** Summary of All Lignin Precursors for LIG Showing Their Raman Ratios, Electrical Properties, and Application^a^

ref	Precursor	Precursor treatment	λ	Laser settings	Atmosphere	*I*_D_/*I*_G_	*I*_2D_/*I*_G_	*R*_s_ (Ω/□)	Application
(124)	KL + PVA	No	10.6 μm	No	Air	0.39	≈0.5^b^	3.8	SC
(127)	LS + PVA, urea	No	10.6 μm	No	Air	0.33	≈0.5^b^	2.8	ES
(125)	KL + PVA	No	10.6 μm	Defocus (best results)	Air	0.37	0.77	4.5	PS
(129)	Lignin + PVA	No	10.6 μm	No	Air	0.36	≈0.6^b^	n.a.	SC
(130)	LS + hydroxyethyl cellulose	FR (B(OH)_3_)	10.6 μm	Multistep Defocus	Air	n.a.	n.a.	3.8	PS
(131)	Alkaline lignin + PEO	No	10.6 μm	No	Air	≈2.2^b^	≈0.5^b^	n.a.	SC
(132)	KL/PEO + PDMS	No	10.6 μm	Defocus	Air	≈2.1^b^	≈0.4^b^	363.1	SC
(133)	Lignin + PES	No	10.6 μm	No	Air	0.5	≈0.5^b^	n.a.	SC
(134)	Lignin + PAN	No	n.a.	No	Air	≈1.1^b^	n.a.	80.5	SC
(136)	Carboxymethyl chitosan + LS on wood	No	10.6 μm	No	n.a.	0.99	n.a.	n.a.	SC
(128)	KL + NaOH, AgNO_3_, PVA	No	800 nm	Defocus	Air	≈0.9^b^	≈0.2^b^	n.a.	ES
(137)	Lignin + PDMS	No	410 nm	No	Air	≈0.5^b^	≈0.5^b^	n.a.	PS
(43)	KL + PDMS	No	355 nm	Defocus	Air	0.53	0.15	n.a.	ES + PS
(138)	Organosolv lignin + carbon cloth	Melting	10.6 μm	No	Air	≈0.4^b^	≈0.8^b^	n.a.	SC
(139)	NanoKL + CNF	No	1030 nm	No	Air	≈0.9^b^	≈0.2^b^	510	n.a.
(140)	NanoKL	No	1030 nm	No	Air	≈0.6^b^	n.a.	306	n.a.
(141)	LS	No	10.6 μm	No	Air	≈1.2^b^	n.a.	1040	PS
(1)	Polyimide	No	10.6 μm	No	Air	1.2	0.7	15	SC

a Acronyms: KL = kraft lignin, PVA = poly(vinyl alcohol), LS = lignosulfonate, PEO = poly(ethylene oxide), PDMS = polydimethylsiloxane, PES = polyethersulfone, PAN = polyacrylonitrile, CNF = cellulose nanofibers, FR = fire-retardant, SC = supercapacitor, ES = electrochemical sensor, PS = physical sensor, n.a. = no value available.

b Extracted from figures because no values were given.

Looking at the three different bioprecursor types, some trends can be identified. The lignocellulosic materials such as wood, cork, and leaves generally show good electrical properties, comparable to PI. These materials can be converted to LIG in an inert atmosphere, by treatment with FR, by short pulse lasers, or by multiple scribing and/or defocus. Combinations of these methods are also feasible. As a general result, a higher lignin content led to an improved performance of the obtained LIG.

Cork can be used directly as a LIG precursor. The native high porosity of cork is preserved in LIG by using lasers with a short wavelength, by boron doping, or by defocused scribing. LIG from cork has good electrical properties. Altogether it is an excellent choice for applications that require a large surface area, such as energy storage or chemical sensing.

LIG from cellulosic materials (with very low lignin content) has poor electrical properties. This is probably related to the large number of structural defects indicated by the higher *I*_D_/*I*_G_ and *I*_2D_/*I*_G_ ratios with respect to other precursors. Furthermore, almost all cellulosic materials could only be turned into LIG with combinations of defocusing, additional FR treatment, inert atmosphere, multistep scribing, or with the addition of extra lignin. This is most likely related to the poor thermal stability of cellulose, as evidenced by thermogravimetric analysis (Figure 8b). Almost all of the mass is lost above 400 °C. Considering the high temperature reached in laser-induced pyrolysis (≈3000 K),^142^ the improved carbonization after the FR treatment makes sense.

Lignin in this respect behaves very differently, with around 40% of its mass retained at 800 °C, making it a much more ideal LIG precursor (Figure 8b). Indeed, LIG from lignin has the best electrical properties with very low sheet resistances down to a few Ω/□. Pyrolysis of lignin did not require any treatment, although defocusing and multistep scribing were sometimes used. However, a relevant disadvantage of lignin is its brittleness, which requires blending it with polymers when used as a film. Moreover, the polymer complexity and diversity (due to source, extraction, and delignification processes)^126^ affect the repeatability of the results. In terms of sustainability, LS are better than the other lignin types because of their good solubility in water, which allows for the avoidance of the use of solvents.

Overall, LIG from all bioderived precursors can match or exceed the performance of LIG from PI, making them an effective and sustainable replacement. In addition to the properties mentioned so far, others such as the surface area and electroactive area, pore dimensions, and wettability come into play when using LIG in applications. For these latter properties, too, bioprecursors represent a valid choice, as will be discussed in detail in the following section.

## 3 Bioderived LIG in Applications

Three main groups of applications for LIG from bioderived precursors can be identified: supercapacitors (SC), electrochemical (ES), and physical sensors (PS). Less frequent applications, not discussed in this section, include circuits from LIG,^91^ electromagnetic field shielding,^99^ triboelectric nanogenerators,^78,84^ touch sensors,^71^ electroluminescence,^71^ and oxygen evolution reactions (O_2_ generation by chemical reactions).^67^ For each of the three main applications, the operative performances of devices obtained from bioderived materials are compared to evaluate the influence of the precursors in the operative life and to assess their differences with respect to the benchmark.

### 3.1 Supercapacitors

**Figure 9:**
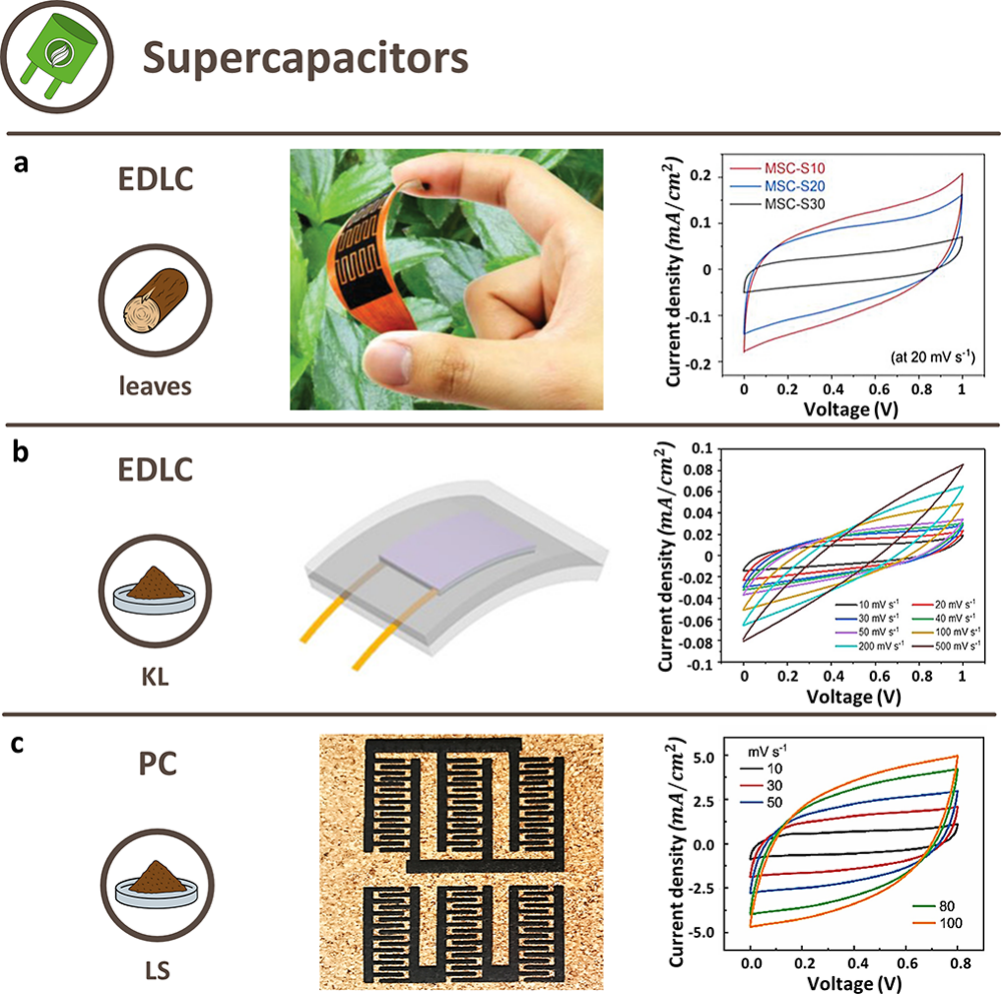
Overview of SC classes from different bioderived LIG precursors. (a) EDLC: representative photograph of an interdigitated EDLC from a leaf-derived LIG with a H_2_SO_4_ /PVA gel electrolyte and CV curves. Reprinted with permission from ref (66). Copyright 2022 Wiley–VCH. (b) Flexible EDLC: schematic illustration of flexible sandwich EDLC from a KL/PEO-derived LIG embedded in PDMS, with a H_2_SO_4_ /PVA gel electrolyte and CV curves obtained at different scan rates. Reprinted with permission from ref (132). Copyright 2020 American Chemical Society. (c) PC: representative photograph of an interdigitated PC from a carboxymethyl chitosan/LS-derived LIG obtained on wood and CV curves at different scan rates. Reprinted with permission from ref (136). Copyright 2022 Elsevier.

SC and microSC are electrochemical energy storage devices based on reversible ions adsorption at the interfaces between electrodes and electrolytes.^144^ They are adopted in electronic systems when high charge–discharge rates, long life cycles, and high power and energy densities are needed.^9^ Two classes of SC can be differentiated based on the charge/discharge mechanism of the device: electric double-layer capacitors (EDLC) (Figure 9a and b) and EDLC with pseudocapacitive behavior (PC) (Figure 9c).^145^ EDLC relies on the physical storage of energy: when the electrodes are immersed in the electrolyte, the ions reorganize spontaneously in a double layer at the interface due to electrostatic attraction. PC, instead, exploits fast and reversible redox reactions happening close to or on the interfaces. In general, EDLC have quicker charge–discharge cycles, longer life cycles, and higher power densities.^144,145^ Other relevant variables that may affect the performance are the pretreatment of the electrodes and the device architecture. The most common are the sandwich, which consists of two thin electrodes on top of each other incorporating an electrolyte layer between them, and the interdigitated, which are arrays of in-plane microelectrode fingers.^146^

Energy storage was one of the first demonstrated applications of LIG from synthetic precursors and is nowadays the focus of about one-third of all the publications published on LIG. Indeed, the intrinsic properties of LIG, i.e., high effective surface area, good porosity, and good conductivity, make it an excellent candidate for developing SC.^1^ A meaningful and accurate comparison among SC with LIG from different precursor materials is very hard since many examples in the literature define specific areal capacitances, while others show the more important specific gravimetric capacitance. However, a list of all bioderived LIG SC and a selection of PI-derived ones (to compare their performance) can be found in Table 6.

**Table 6:** Summary of all SC applications of different bioderived precursors in comparison with SC made from PI-derived LIG^a^

ref	Precursor	λ	SC class	Electrode design	Electrolyte	Treatment	*C*_a_ (mF/cm^2^)	Scan rate/current density	ESR (Ω)
(66)	Leaves	346 nm	EDLC	Interdigitated	H_2_SO_4_/PVA	none	34.68	5 mV s^–1^	132.5
(72)	Bamboo	522 nm	EDLC	Interdigitated	agarose/NaCl	none	2.8	n.a.	n.a.
(76)	Agglomerated cork	1.06 μm	EDLC	Interdigitated	H_2_SO_4_/PVA	none	1.43	0.1 mA cm^–2^	n.a.
(94)	Office paper	10.6 μm	EDLC	Interdigitated	H_2_SO_4_/PVA	Ag coating and UV hydrophilization	4.6	0.015 mA cm^–2^	n.a.
(124)	KL + PVA	10.6 μm	EDLC	Interdigitated	H_2_SO_4_/PVA	Au sputtering	25.1	0.05 mA cm^–2^	8.5
(131)	Alkaline lignin + PEO	10.6 μm	EDLC	Sandwich	H_2_SO_4_/PVA	none	25.44	0.1 mA cm^–2^	n.a.
(132)	KL/PEO + PDMS	10.6 μm	EDLC	Sandwich	H_2_SO_4_/PVA	transfer onto PDMS	2.51	0.01 mA cm^–2^	n.a.
(133)	Lignin + PES	10.6 μm	EDLC	Sandwich	H_2_SO_4_/PVA	none	11	0.05 mA cm^–2^	n.a.
(138)	Organosolv lignin + carbon cloth	10.6 μm	EDLC	Sandwich	H_2_SO_4_/PVA	none	149.4	0.5 mA cm^–2^	3.3
(19)	Wood	10.6 μm	PC	n.a.	H_2_SO_4_/PVA	PANI electrodeposition	780	1 mA cm^–2^	6
(68)	Wood	346 nm	PC	Sandwich	Na_2_SO_4_	KMnO_4_	53.6	1 mA cm^–2^	n.a.
(77)	Cork	450 nm	PC	Sandwich	H_2_SO_4_/PVA	boric acid	11.24	0.1 mA cm^–2^	11.13
(98)	MCG	10.6 μm	PC	Interdigitated	LiTFSI/PVA	Mo/hydrogel coating	14	1 mV s^–1^	n.a.
(129)	Lignin + PVA	10.6 μm	PC	Interdigitated	H_2_SO_4_/PVA	Ti_3_C_2_T_*x*_/CuFe-PBA	198	1 mA cm^–2^	4.5–5
(136)	Carboxymethyl chitosan + LS on wood	10.6 μm	PC	Interdigitated	H_2_SO_4_/PVA	none	82.1	0.1 mA cm^–2^	33.91
(134)	Lignin + PAN	n.a.	PC	Interdigitated	H_2_SO_4_/PVA	MoS_2_ coating	16.2	0.1 mA cm^–2^	113
(1)	PI	10.6 μm	EDLC	Interdigitated	H_2_SO_4_	none	4	0.2 mA cm^–2^	7
(153)	PI	10.6 μm	EDLC	Sandwich	H_2_SO_4_/PVA	none	9	0.02 mA cm^–2^	n.a.
(147)	PI	10.6 μm	PC	Interdigitated	PVA/LiCl/PVA/H_2_SO_4_	MnO_2_/FeOOH/PANI electrodeposition	361	0.5 mA cm^–2^	n.a.
(154)	PI	10.6 μm	PC	n.a.	PVP/NaCl	MoS_2_ coating	0.014	0.1 mA cm^–2^	102
(155)	PI	10.6 μm	EDLC	Sandwich	H_2_SO_4_/PVA	PAA coating + second carbonization	19.8	0.05 mA cm^–2^	≈10
(156)	PI	10.6 μm	PC	Interdigitated	H_3_PO_4_ /PVA	CoCl_2_ + gelatin	22.3	0.05 mA cm^–2^	n.a.
(157)	PI/KOH	10.6 μm	EDLC	Interdigitated	H_3_PO_4_ /PVA	KOH	32	0.05 mA cm^–2^	120–170
(158)	PI/sodium CMC	10.6 μm	EDLC	Interdigitated	H_2_SO_4_/PVA	boron-doped	60.6	0.08 mA cm^–2^	7.2
(159)	PI/H_3_BO_3_	10.6 μm	PC	Sandwich	H_2_SO_4_/PVA	H_3_BO_3_/PAA coating + second carbonization	40.4	0.05 mA cm^–2^	5.8

a Acronyms: SC = supercapacitor, *C*_a_ = specific areal capacitance, ESR = equivalent series resistance, EDLC = electric double layer SC, PVA = poly(vinyl alcohol), KL = kraft lignin, PEO = poly(ethylene oxide), PDMS = polydimethylsiloxane, PES = poly(ether sulfone), PC = hybrid SC with pseudocapacitive contribution, MCG = molybdenum carbide-graphene, PBA = Prussian blue analogue, LS = lignosulfonate, PAN = polyacrylonitrile, PI = polyimide, PANI = polyaniline, PVP = poly(vinylpyrrolidone), n.a. = no value available.

#### Electric Double-Layer Capacitor

Among lignocellulosic LIG precursors, leaves had the best areal capacitance (*C*_a_ = 34.68 mF cm^–2^ at 5 mV s^–1^), with H_2_SO_4_/PVA as electrolyte and interdigitated electrodes.^66^ With the same electrode architecture, bamboo-^72^ and paper-derived^94^ LIG performed worse, with *C*_a_ = 2.8 mF cm^–2^ and 4.6 mF cm^–2^, respectively. Cork-derived LIG was the worst SC of this group, with a slightly lower areal capacitance.^76^ Apart from the electrolytes (agarose hydrogel containing NaCl^72^), no major differences in the architecture of these SC are reported, thus the main influence factors may be ascribable to the lignin content of the precursors and laser source/scribing parameters adopted to obtain LIG.

Many examples of EDLC made of lignin-derived LIG electrodes are reported, all with H_2_SO_4_/PVA as the electrolyte, with either interdigitated or sandwich structures. In one case, the capacitance was increased from *C*_a_ = 17.0 mF cm^–2^ at 0.05 mA cm^–2^ by 1.4 times by sputtering Au on top of the electrodes. This increase was related to the increase in the conductivity of the electrodes.^124^ By coating the LIG, the capacitance at low charge–discharge rates increased, probably due to the increased utilization of meso/macropores attributed to better charge carrier distribution in the horizontal direction,^124^ while at higher current densities the capacitance decreased because the Au coating covered the surface macropores of the electrodes. It was also reported that the areal capacitance decreased with the laser power increase because of the fewer and smaller nanopores and the unsuitable morphology of LIG.

Also, untreated lignin has been successfully employed for EDLC: the best result was lignin powder melted onto a carbon cloth and then scribed to make sandwich LIG electrodes, a peculiar approach that lead to a *C*_a_ = 149.4 mF cm^–2^ at 0.5 mA cm^–2^ and a very low equivalent series resistance (ESR) of 3.3 Ω.^138^ XPS analysis showed that a higher number of oxygen groups in the LIG electrodes enhanced the adsorption of the electrolyte and therefore improved the capacitance. Another significant example showed a *C*_a_ = 25.44 mF cm^–2^ at 0.1 mA cm^–2^ obtained from lignin/PEO precursor, which is similar to the result obtained by^124^ but without the need for pretreatment, probably due to the different precursor.^131^ Slightly worse results have been obtained with lignin/PES, which however showed a better result than PES alone, with *C*_a_ = 11 mF cm^–2^ against *C*_a_ = 0.69 mF cm^–2^, at 0.05 mA cm^–2^, and higher cycling stability.^133^

An interesting example of LIG SC for portable/wearable applications was a highly flexible EDLC obtained from LIG based on a KL/PEO composite, embedded in PDMS (Figure 9b).^132^ It showed the ability to endure bending deformation with little or no degradation of its capacitance, which however was worse than other LIG EDLC.^132^

#### Electric Double-Layer Capacitor with Pseudocapacitive Behavior

For PC, most of the approaches involved a treatment to introduce pseudocapacitive behavior. Almost all the SC adopted H_2_SO_4_/PVA as electrolyte, but also LiTFSI/PVA^98^ and Na_2_SO_4_.^68^ The specific electrolyte did not seem to be the most affecting parameter on their performances. The highest capacitance (*C*_a_ ≈780 mF cm^–2^ at 1 mA cm^–2^) and a good ERS of ≈6 Ω were obtained thanks to the electrodeposition of polyaniline (PANI) onto LIG patterned on pine wood.^19^ These values were considered very promising and comparable with those obtained for PI-derived LIG and PANI.^147^ The very low C_a_ on pure LIG (*C*_a_ ≈1 mF cm^–2^ at 1 mA cm^–2^) was similar to the one obtained from pure PI-derived LIG,^1^ demonstrating that the areal capacitance can be greatly improved by depositing pseudocapacitive materials onto the LIG, independently of the precursor material. Another example of PC from a lignocellulosic material was a sandwich SC made of LIG from a KMnO_4_-soaked wood, which showed an improvement in *C*_a_ from ≈3.5 mF cm^–2^ to ≈53.6 mF cm^–2^ at 1 mA cm^–2^, for the untreated and treated LIG precursor, respectively.^68^ Cork was treated with boric acid to obtain LIG electrodes, for both interdigitated and sandwich SC having PC behavior.^77^ The treatment improved the *C*_a_ around three times, up to 11.24 mF/cm^2^ at 0.1 mA/cm^2^. The increased performance was attributed to the catalytic effect of boron doping, which resulted in a higher oxygen concentration in the LIG, as proved by XPS. Furthermore, it was stated that the boron doping “can induce hole charge carriers in the graphene lattice, resulting in an increase of the charge density and hence the electrons’ charge storage”.^77^

Cellulosic precursors (i.e., MCG) for PC electrodes have been reported too, with significantly lower performances than those discussed so far.^98^ The paper was treated with Mo_3_C_2_ prior to lasing, giving the PC behavior to the SC, similar to what was reported for boron doping.^77^

Instead, lignin-derived LIG showed two of the best performances for PC. A lignin/PVA composite was used as LIG precursor for an interdigitated SC.^129^ One of the arrays was spray-painted with MXene (Ti_3_C_2_T_*x*_ flakes), to achieve high pseudocapacitance,^148^ while the other was spray-coated with a CuFe-Prussian blue analogue (PBA) ink, to improve energy storage and conversion ability.^149^ Both coated electrodes showed an increase in areal capacitance with an increasing mass loading. A SC with just Ti_3_C_2_T_*x*_ was investigated with a gel electrolyte of H_2_SO_4_/PVA. The good performance was attributed to the 3D conductive network of LIG and the fast redox kinetics of Ti_3_C_2_T_*x*_ MXene.^129^ The hybrid PC showed a very low ESR = 4.5–5 Ω, independent of the increase in mass loading. The device showed a maximum *C*_a_ = 198 mF cm^–2^ at 1 mA cm^–2^; which increased linearly with the increase of mass loading. A composite of carboxymethyl chitosan and LS was applied on a wood substrate to create a PC with heteroatom doping.^136^ By increasing the mass of the coating, a capacitance around 4 times larger than the uncoated wood precursor was obtained. The good performance of this SC was attributed to the porous structure with micro-, meso-, and macropores in addition to the conductivity of LIG and the pseudocapacitive properties given by heteroatom doping (O, N, and S, coming from the coating). Another possible treatment was MoS_2_ nanosheet doping of a lignin/PAN precursor, prior to LIG scribing, which allowed the capacitance to improve.^134^ This was ascribed to the higher electrical conductivity of MoS_2_ and to the new interfaces available for ion intercalation, but they were lower than those of the other lignin precursors.

**Figure 10:**
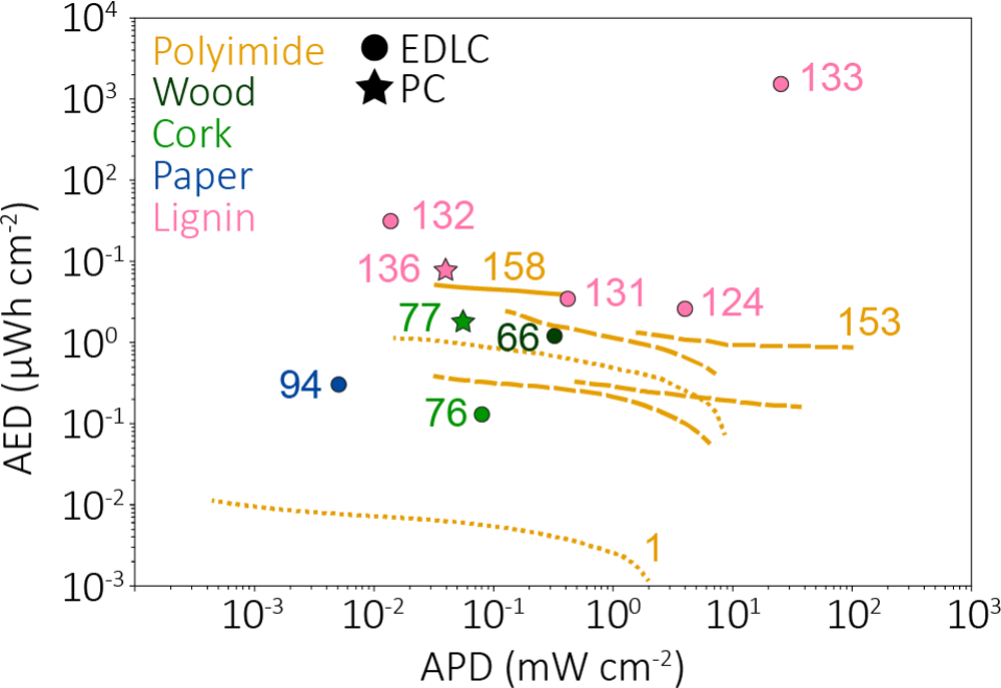
Ragone plot for AED and APD comparing the bioderived LIG (dots for EDLC and stars for PC) to the PI-derived LIG (lines) SC.

Comparing the areal capacitance and treatment of the bioderived LIG with PI-derived LIG SC shows that they perform similarly or better (see Table 6). These conclusions are summarized in the Ragone plot (Figure 10), in which the bioderived (dots/stars) overlap with PI-derived (lines) SC. Interestingly, some SC (both EDLC and PC) from bioderived precursors show particularly good performances without any additional treatment. However, no rationalization for this exceptional behavior is given, and thus it can be supposed that it is related to a combination of factors: the precursor composition, electrode arrangement, laser type, or electrolyte-LIG interaction. While wood-derived SC show the highest areal capacitance, lignin-derived SC had the best performances in terms of both areal energy density (AED) and areal power density (APD). Lignin-derived LIG could therefore be considered the most appropriate candidate for SC. Cellulosic materials were rarely included in SC investigations because of their higher sheet resistance, which strongly influences the APD.^150^ It is, however, important to remark that no definite statement can be made because not all publications stated their APD and AED. AED can be significantly increased by “optimizing the pore size of carbon electrodes to match a given electrolyte”.^151^ Generally, it can be said that the AED “is a nonmonotonic function of the pore width”, whose maximum depends on the voltage and is related to the optimal pore size. If the pores have similar diameters, the stored energy density further increases.^152^ This optimal size is also connected to the ion size and directly proportional to the operating voltage. However, it was reported that a broad pore size distribution can significantly decrease or even erase the connection with capacitance; hence, a tight size distribution is required for good energy storage. Even if characterizing the porosity of a precursor provides interesting information for the performance analysis, porosity itself is not enough to define a material: indeed, different materials with the same porosity can result in completely different capacitive properties.^152^ Also, other features and important characteristics come into play depending on the specific application (even if in this case they were mainly LCD^66,68,136^ and LED^72,94^), such as the high flexibility of devices in some cases.

### 3.2 Electrochemical Sensors

**Figure 11:**
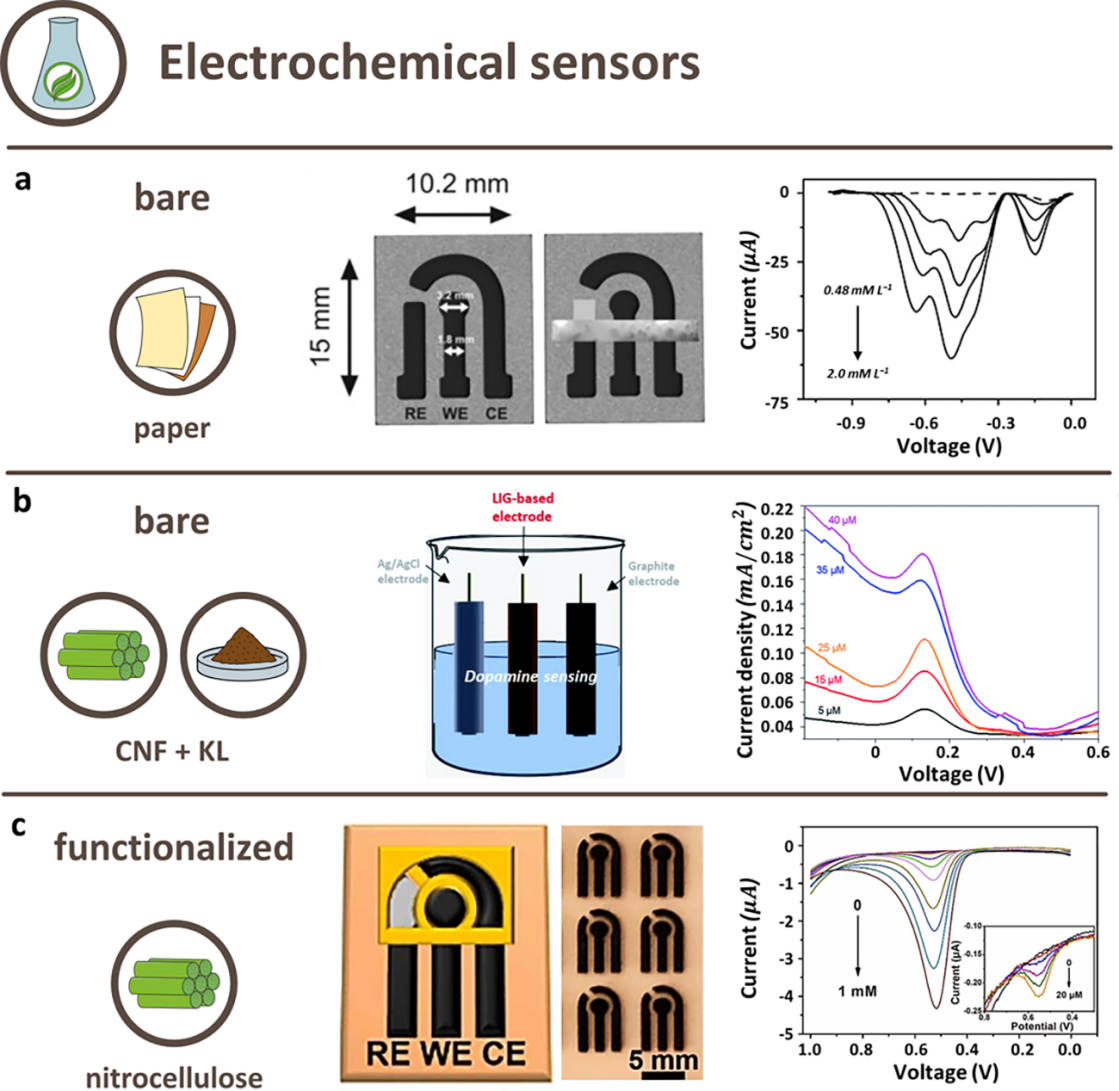
Overview of the ES applications from different bioderived LIG precursors. (a) Schematic illustration of a bare ES from a paperboard-derived LIG with Ag ink pattern and differential pulse voltammetry curves, obtained in a 0.1 M L^–1^ PBS (pH 2.0) (- - -) and in a 0.48, 0.91, 1.30, and 2.0 mM L^–1^ picric acid solution (—). Reprinted with permission from ref (20). Copyright 2017 Wiley–VCH. (b) Schematic illustration of a bare ES from a CNF/KL-derived LIG and differential pulse voltammetry curves for 5–40 μM dopamine solutions. Reprinted with permission from ref (115). Avilable under a CC BY-NC 3.0 license. (c) Schematic illustration of a functionalized ES from a nitrocellulose-derived LIG and differential pulse voltammetry curves of 0–1000 μM NaNO_2_ on the sensor (inset shows curves of 0–20 μM NaNO_2_). Reprinted with permission from ref (119). Copyright 2022 Elsevier.

Most sensors are manufactured with a three-electrode design, i.e., working (WE), counter (CE), and Ag ink-coated reference electrodes (RE), apart from refs (43) and^127^ using a different design). Two main classes are highlighted based on the WE: bare (Figure 11a and b) and functionalized (Figure 11c) electrodes, with modifications that range from biomolecules (such as enzymes, aptamers, and antibodies), to metal nanoparticles (Cu, Au, Pt, Pd) and polymer coating.^10^ The performances of bioderived LIG sensors and some PI-derived LIG sensors (taken as references) are compared in terms of lower detection limits, detection ranges, and sensitivity (Table 7).

**Table 7:** Summary of All ES Applications of LIG from Different Bioderived Precursors^a^

ref	Precursor	λ	Analyte	Transduction method	Functionalization method	Detection limit	Dynamic range	Sensitivity
(20)	Paperboard	10.6 μm	Ascorbic acid	CV	None	n.a.	0–5.0 mM L^–1^	n.a.
(86)	Paperboard	10.6 μm	Caffeic acid	CV	None	n.a.	0.91–2.86 mM L^–1^	n.a.
(87)	Paperboard	10.6 μm	Picric acid	CV	None	n.a.	0.48–2.0 mM L^–1^	n.a.
(98)	Paperboard	10.6 μm	Sulfites in commercial beverages	CV	None	1.2 mg L^–1^	2.5–25 mg L^–1^	n.a.
(88)	Paperboard	10.6 μm	Lead in water	SWV	None	6 μg L^–1^	Up to 50 μg L^–1^	n.a.
(93)	Office paper MCG	10.6 μm	Cu^2+^	LSV	None	10 μM L^–1^	0.01–10 mM L^–1^	n.a.
(62)	Office paper	10.6 μm	Furosemide	DPV	None	24 μM L^–1^	25–196 μM L^–1^	n.a.
(115)	Filter paper	10.6 μm	Uric acid	CV	None	3.97 μM	10–300 μM	0.363 μA cm^–2^ μM^–1^
(97)	Filter paper	10.6 μm	Uric acid	SWV, CA	None	41 nM	1–1000 μM	24.35 μA μM^–1^
(119)	CNF and KL	10.6 μm	Dopamine	DPV	None	3.4 mM	5–40 μM	4.39 μA μM^–1^ cm^–2^
(127)	Filter paper Office paper	10.6 μm	Glucose	CA	Enzymes	0.13 mM	Up to 1 mM	27.24 μA mM^–1^
(128)	Nail polish (nitrocellulose	405 nm	Nitrite	DPV	Chitosan	0.9 μM	2.0–1000 μM	n.a.
(43)	Nail polish (nitrocellulose	405 nm	Glucose	DPV	CuCl_2_	50 μM	0.1–10 mM	n.a.
(164)	LS + PVA, urea	10.6 μm	Glucose	CA	Enzymes + Ti_3_C_2_T_*x*_/PB + chitosan	0.3 μM	10 μM-5.3 mM	49.2 μAmM^–1^ cm^–2^
(161)	LS + PVA, urea	10.6 μm	Lactate	CA	Enzymes + Ti_3_C_2_T_*x*_/PB + chitosan	0.5 μM	up to 20 mM	21.6 μA mM^–1^ cm^–2^
(167)	LS + PVA, urea	10.6 μm	Alcohol	CA	Enzymes + Ti_3_C_2_T_*x*_/PB + chitosan	0 μM	0–50 mM	5.78 μA mM^–1^ cm^–2^
(168)	KL + NaOH, AgNO3, PVA	800 nm	Nitrite	DPV, CA	Ag nanoparticles	117 nM,	1–600, 600–4000 μM	277.7 μA mM^–1^ cm^–2^
(160)	KL + NaOH, AgNO3, PVA	800 nm	Dopamine	DPV, CA	Ag nanoparticles	98 nM	1–5 μM	788.9 μA mM^–1^ cm^–2^
(43)	KL lignin + PDMS	355 nm	Sweat ion Na	P	Ion-selective membrane	n.a.	100 nM to 0.1 M	63.6 mV dec^–1^
(43)	KL lignin + PDMS	355 nm	Sweat ion K	P	Ion-selective membrane	n.a.	10 nM to 0.1 M	59.2 mV dec^–1^
(164)	PI	10.6 μm	Uric acid	DPV	None	0.74 μM	3–40 μM	3.50 μA μM^–1^ cm^–2^
(161)	PI	10.6 μm	Dopamine	CV, DPV	None	0.50 μM	0.5–3 μM	93 μA μM^–1^ cm^–2^
(161)	PI	355 nm	Dopamine	CV, DPV	None	0.50 μM	0.5–4 μM	58 μA μM^–1^ cm^–2^
(167)	PI	n.a.	Glucose	CV	Cu nanoparticles	0.4 μM	1 μm to 6.0 mM	495 μA mM^–1^ cm^–2^
(168)	PI	10.6 μm	Glucose	CV	Pt nanoparticles and chitosan with glucose oxidase	0.3 μM	0.3 μM to 2.1 mM	65.6 μA mM^–1^ cm^–2^
(160)	PI	10.6 μm	Dopamine	CV	Pt nanoparticles	0.07 μM	n.a.	6995 μA mM^–1^ cm^–2^
(160)	PI	10.6 μm	Ascorbic acid	CV	Pt nanoparticles	6.10 μM	n.a.	250.7 μA mM^–1^ cm^–2^
(160)	PI	10.6 μm	Uric acid	CV	Pt nanoparticles	0.22 μM	n.a.	8289 μA mM^–1^ cm^–2^
(169)	PI	10.6 μm	Ascorbic acid	CV	Pyrrole	n.a.	1.5–4 mM	1.356 μA/decade
(169)	PI	10.6 μm	Amoxicillin	CV	Eriochrome black T	11.98 nM	50 nM to 100 μM	–13.32 μA/decade

a Acronyms: CV = cyclic voltammetry, DPV = differential pulse voltammetry, SWV = square wave voltammetry, LSV = linear sweep voltammetry, CA = chronoamperometry, *P* = potentiometry, MCG = molybdenum carbide-graphene, CNF = cellulose nanofibers, KL = kraft lignin, LS = lignosulfonate, PVA = poly(vinyl alcohol), PDMS = polydimethylsiloxane, PI = polyimide, n.a. = no value available.

#### Bare LIG

Many bare electrodes from cellulosic LIG precursors are reported in the literature, all scribed with a 10.6 μm CO_2_ laser. Different kinds of papers (i.e., paperboard,^20,86,87^ office paper^88,98^), filter paper,^62,93^ and CNF combined with KL^115^ were adopted to detect analytes for different application fields, such as clinical,^62,93,115^ food,^20,86^ and forensic.^20^ Among the paperboard-derived LIG sensors, one showed high conductivity and a good active area to geometric area ratio of 6.5.^20^ SEM and EDX analyses revealed that the LIG was decorated with aluminosilicate nanoparticles, coming from the kaolin filling of the paper precursor. This may be connected to the resistance of the equivalent circuit obtained via electrochemical impedance spectroscopy, which was lower than that of glassy carbon or screen-printed carbon electrodes. The sensor has been used for the detection of ascorbic acid and caffeic acid, which are important antioxidants present in foods and nutritional supplements, and for the forensic detection of picric acid. Another similar sensor was instead exploited for sulfite detection in commercial beverages.^86^ A method called gas-diffusion microextraction was used to avoid the need for surface modification. The third example used a preconcentration of the metal cations on the surface of the WE.^87^ The cations were reduced and dissolved in the solution and then oxidized back on the electrode with potential sweeping. The metals could be recognized with different oxidation peaks. The voltammetric behavior shows a semi-infinite linear diffusion caused by thin-layer effects within the pores and diffusion to the top electrode layer.^61^ The charge transfer process could be limited by the transport resistance, as shown by electrochemical impedance spectroscopy evaluation. The increased electroactive area was attributed to the rough and porous structure, which could correlate with the high-density defect regime of LIG.^61^

Pretreated (Mo^5+^ hydrogel^98^ and pencil^88^) office paper LIG electrodes were also tested. They were used to detect heavy ions (Cu^2+^) in H_2_SO_4_ solutions^98^ and furosemide, a diuretic drug, in synthetic urine.^88^ The sensor was also preliminarily tested for gas sensing on polar molecules, such as moisture and methanol. Instead, LIG electrodes obtained from pencil-treated office paper had better electrochemical performances than untreated office paper and were comparable with screen-printed graphene electrodes. Measurements in synthetic urine showed that the sensor could detect furosemide in concentrations ranging from 25 to 196 mM L^–1^. Interference studies with ascorbic acid, uric acid, urea, creatinine, and glucose were performed, and it was shown that urea had the strongest influence on sensing.^88^

Filter paper LIG electrodes had applications with two devices for nonenzymatic detection of uric acid in urine.^62,93^ The first^93^ was able to detect and quantify uric acid in a buffer solution. Selectivity tests with ascorbic acid and dopamine showed that there were some interferences, and fouling occurred at the electrode. However, the concentrations at which the problems occurred were so high that after dilution (1:20) of the urine sample such values were hardly ever found in real urine samples. A very low electron transfer rate constant of *k*_0_ = 1.4 × 10^–3^ cm s^–1^ was measured which was smaller than PI-derived LIG.^160,161^ The second^62^ consisted in a systematic in-depth study of LIG synthesis to elucidate the complex relationship between surface microstructure and resulting electroanalytical properties. The sample with multistep laser scribing (one defocused and one focused) showed the best sensing performances, due to better LIG crystallinity and lower resistance.

The last class of cellulosic LIG for electrochemical sensing is a combination of CNF and KL to detect dopamine in a buffer solution.^115^ Compared to a glassy carbon electrode, the LIG electrode showed a significantly improved sensitivity (4–5 times). However, the lowest detection limit (out of linear range) was higher than those for functionalized LIG electrodes (Table 7).

#### Functionalized LIG

Functionalized LIG electrodes for ES were again mostly obtained from cellulosic materials^97,119^ and lignin^43,127,128^ scribed with different lasers, all for applications in the clinical and food fields. Cellulosic precursor materials included filter and office paper^97^ and nitrocellulose from nail polish.^119^ They were both exploited for glucose monitoring in phosphate-buffered saline (PBS), and the second was also exploited for nitrite. Electrodes for enzymatic glucose sensing were obtained by scribing on wax-coated FR-treated paper and then functionalizing the resulting LIG with a solution of graphene oxide, peroxidase, and D-trehalose.^97^ A 2-fold increase in the electroactive surface area to geometrical area ratio was achieved for filter paper compared to office paper. The increase was related to the higher surface area created by the carbonized natural fiber network. A better performance of the filter paper was attributed to the natural fiber network structure and the higher conductivity of the obtained LIG. The nitrocellulose-derived LIG electrode was exploited for nonenzymatic glucose monitoring, by mixing the nitrocellulose with CuCl_2_ before laser scribing.^119^ The resulting LIG was doped with Cu^2+^, as proven by SEM and EDX. A strong oxidation peak is observed for glucose, due to the catalytic oxidation of glucose, and the sensor showed acceptable selectivity toward typical interferents.^97^ Investigations of the electrochemical performance for nitrite detection showed that the bare LIG electrode had much lower electrochemical activity than PI-derived LIG, probably due to the lower amount of defects.^61^ The WE was coated with chitosan because of its ability to promote the response of nitrite via electrostatic accumulation and to improve the electrode selectivity. The sensor was used to measure the concentration of nitrite in tap water and lake water. Furthermore, the authors showed that LIG obtained from different compositions/brands of nail polish did not influence the sensor’s performance.

Different functionalized electrodes were obtained from lignin, both KL^43,128^ and LS.^127^ One electrode was functionalized with Ag nanoparticles, which were created during the lasering process due to the incorporation of AgNO_3_ in the precursor.^128^ With an increasing AgNO_3_ concentration, the redox current increased because of the reduced resistance of the LIG with more nanoparticles. The LIG electrodes were used to detect nitrite and dopamine in solution. Selectivity tests showed that there was only a small current response to the addition of ascorbic acid and no response to the addition of uric acid, indicating that there is not much or no interference from these species. A specific five-electrode design was developed for simultaneous multianalyte (glucose, lactate, and alcohol) sensing in an artificial sweat solution,^127^ and resulted in a user-friendly, low-cost and sustainable approach to avoid material waste and allow for an easier sweat collection procedure. The WE were modified with MXene/PBA (Ti_3_C_2_T_*x*_/PBA) by a spraying-coating process to significantly increase the dynamic range and sensitivity for all three analytes, due to the improved electrochemical H_2_O_2_ detection capability compared to graphene/PBA and carbon nanotubes/PBA composites.^127^ The sensors were then assembled by immobilizing the catalytic enzymes, i.e., glucose oxidase, lactate oxidase, and alcohol oxidase, for the detection of glucose, lactate, and alcohol, respectively. A different approach was the functionalization of the electrodes with an ion-selective membrane made of ionophore, plasticizer, lipophilic additive, and matrix, to avoid any internal reference solution.^43^ The ionophore allowed the selective measurement of specific ions, while the lipophilic additive drew the ions toward the membrane with its opposite charge; the plasticizer and matrix were needed for good dispersion of the other components. The sensor was specifically designed with two WE and an RE to simultaneously detect Na and K cations in sweat with a good response time.

The performance of cellulose- and lignin-derived LIG in ES is comparable to PI-derived LIG ones^10^ and to ES in general,^160,162^ in terms of detection limit, range and sensitivity (Table 7). A case study on dopamine to compare bare and functionalized bioderived LIG electrodes and bare and functionalized PI-derived LIG electrodes showed that the detection limit and sensitivity were comparable for all the samples.^115,128,160,161^ Similar conclusions can be drawn if other target molecules are considered, such as uric acid^97,115,154,163^ and glucose.^19,43,127,155,164^ Further differences within the same LIG precursor can come from the precursor itself, the pretreatment, or the laser source. Moreover, surface contaminants that can influence the sensing performance of electrochemical sensors can be introduced at any step of production regardless of the precursor. However, a major effect of surface oxygen groups was observed in literature even for functionalized electrodes.^165^ These oxygen groups are inherent to the laser-induced pyrolysis and originate from both the atmosphere and the precursor.

Notably, glucose detection was performed only with functionalized electrodes, probably because graphene is not reactive enough to this molecule.^166^ ES with lignocellulosic-derived LIG are not reported in the literature: even if the porous structure could be an advantage for electrochemical sensing, it may be deduced that the raw nature of these materials makes them a poor choice for thin and flexible applications, such as needed for many ES applications and in particular for wearables. However, future studies may be useful to evaluate their electrochemical performances for different applications.

### 3.3 Physical Sensors

**Figure 12:**
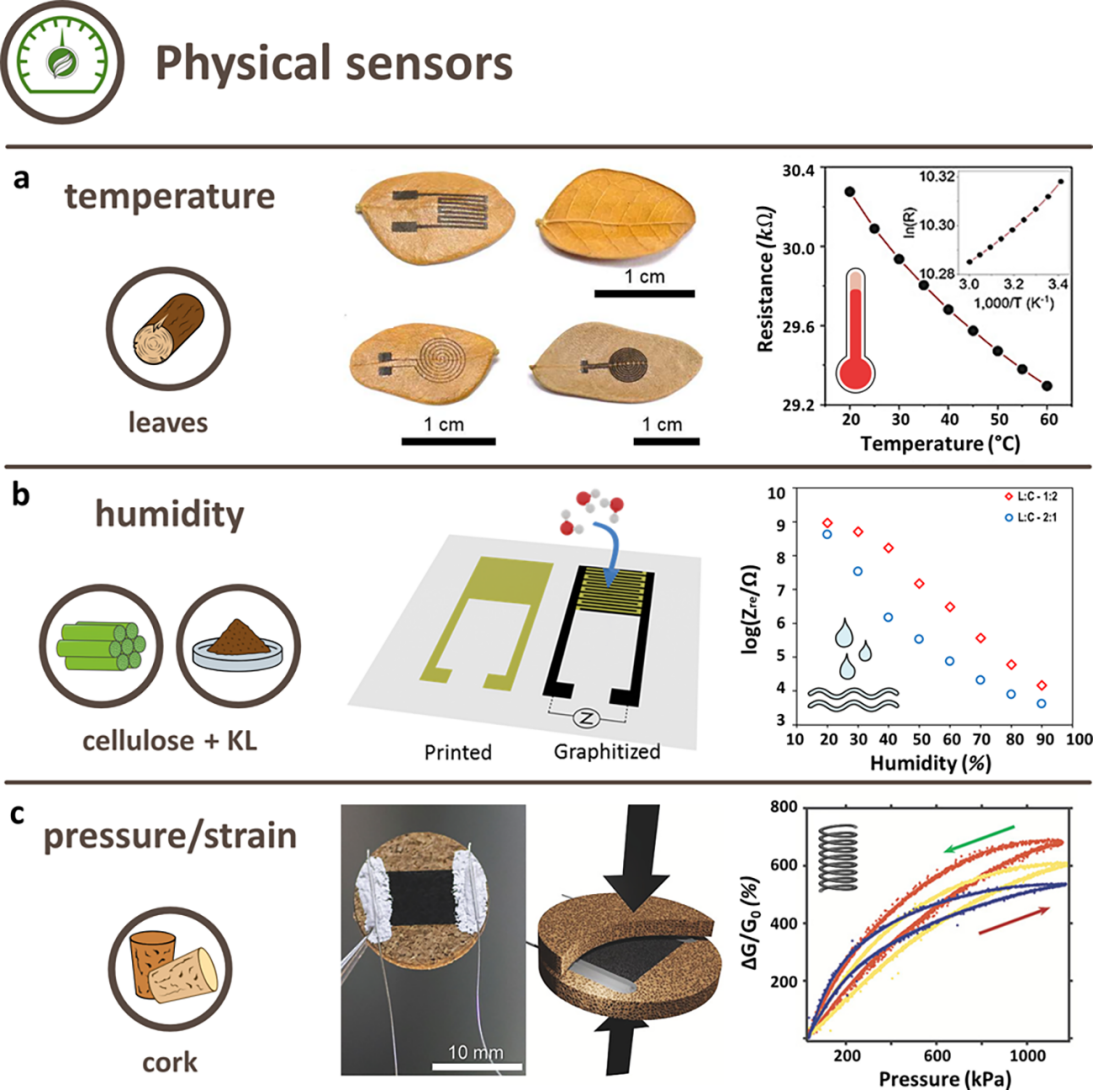
Overview of the main PS applications from different bioderived LIG precursors: (a) temperature, (b) humidity, and (c) strain/pressure sensors. (a) Representative photograph of temperature sensor from a leaf-based LIG and resistance variation as a function of temperature, indicating a negative temperature coefficient behavior (the inset shows the dependency of ln(*R*) on 1/*T*). Reprinted with permission from ref (68). Copyright 2019 Wiley–VCH. (b) Schematic of the humidity sensors from a LS/cellulose-derived LIG and real part impedance (*Z*_re_) data taken at 10 Hz. Reprinted with permission from ref (130). Available under a CC BY 4.0 license. (c) Representative photograph of piezoresistive pressure sensor from a cork-derived LIG and relative conductance versus pressure (the red and green arrows represent the loading and unloading sides of the cycle, respectively). Reprinted with permission from ref (79). Copyright 2020 Wiley–VCH.

LIG-based PS is able to measure a variety of parameters, including strain, pressure, bending, humidity, and temperature. They are of high relevance in several application fields, such as healthcare for humans and animals (e.g., heart rate, pulse monitoring, plantar pressure), environmental monitoring (e.g., temperature detection, gas sensing, pressure and humidity sensing), and robotics (e.g., tactile sensing, gesture-based control, sound detecting, proximity, and positioning).^170^ The possibility of using bioderived LIG precursors is particularly interesting for PS because of the possible applications for transient electronics, such as soil moisture monitoring^171^ and bioresorbable pressure sensing.^172^ This section takes a look at the different types of PS from bioderived LIG and compares them to the performance of PI-derived LIG sensors (Table 8). Three main applications are highlighted: temperature (Figure 12a), humidity (Figure 12b), and strain/pressure sensing (Figure 12c).

**Table 8:** Summary of All PS Applications of LIG from Different Bioderived Precursors Showing the Type and Sensitivity of the Sensor^a^

ref	Precursor	λ	PS type	Sensitivity
(68)	Leaves	343 nm	Temperature	α = –8 × 10^–4^ °C^–1^
(96)	Filter paper	355 nm	Temperature	α = –2.8× 10^–3^ °C^–1^
(83)	Colored paper, paper cup	532 nm	Temperature	α = –1.5 × 10^–3^ °C^–1^
(100)	Cardboard	532 nm	Temperature	α = –2 × 10^–3^ °C^–1^
(95)	Carboxymethyl Xylan (hemicellulose)	10.6 μm	Temperature	α = –1.29 Ω °C^–1^
(96)	Filter paper	355 nm	Humidity	1.3 × 10^–3^ %RH^–1^
(100)	Cardboard	532 nm	Humidity	36.75 fF %RH^–1^
(130)	LS + hydroxyethyl cellulose	10.6 μm	Humidity	n.a.
(141)	LS	10.6 μm	Humidity	0.0144%RH^–1^
(71)	Wood	10.6 μm	Strain, bending	n.a.
(79)	Cork	355 nm	Pressure	up to 9.8 × 10^–3^ kPa^–1^ , average <600 kPa
(100)	Cardboard	532 nm	Pressure	≈ −0.563 kPa^–1^ for 0.009–50 kPa^–1^
(92)	Filter paper	10.6 μm	Strain, bending	GF ≈ 42
(85)	Office paper MCG	10.6 μm	Strain, pressure	GF = 73 (tensile), GF = 43 (compression)
(141)	LS	10.6 μm	Force	GF = 60–180
(137)	Lignin + PDMS	410 nm	Pressure	n.a.
(125)	KL + PVA	10.6 μm	Strain	GF = 100–960 for ε = 0–14%
(43)	KL + PDMS	355 nm	Strain	GF ≈ 20
(90)	Paperboard	10.6 μm	NH3 gas sensor	n.a.
(83)	Colored paper	532 nm	TMA gas sensing	gas coefficient of *R* = 0.0041% ppm^–1^
(116)	CNC	10.6 μm	UV Photodetectors	1 μA/W
(173)	PI	1064 nm	Temperature	α = 1.24× 10^–3^ °C^–1^
(174)	PI	10.6 μm	Temperature	α = 0.97 × 10^–2^ °C^–1^
(184)	PI + GO	n.a.	Humidity	4770.14 pF %RH^–1^
(185)	PI	450 nm	Humidity	3215.25 pF %RH^–1^
(179)	PI + PDMS	10.6 μm	Strain	GF = 50–20 000
(180)	PI	355 nm	Strain	GF = 20
(181)	PI + MoS_2_	10.6 μm	Strain	GF ≈ 1242
(186)	PI	10.6 μm	Strain	GF = 10–38
(187)	PI + PDMS	10.6 μm	Strain	GF = 15.79
(188)	PI + Ecoflex + silver	10.6 μm	Strain	GF = 223.6
(189)	PI + Fe_2_O_3_	10.6 μm	Pressure	603 kPa^–1^ for 0–10 kPa
(190)	PI + rGO cloth	10.6 μm	Pressure	30.3 kPa^–1^ for 0–2.5 kPa
(191)	PDMS + LIG paste	10.6 μm	Pressure	1.86 kPa^–1^ up to 150 Pa
(192)	PI + PU + PS spheres	10.6 μm	Pressure	2048 kPa^–1^ for 10–100 kPa
(193)	PDMS	405 nm	Pressure	∼480 kPa^–1^ for 0–100 Pa

a Acronyms: PS = physical sensor, LS = lignosulfonate, MCG = molybdenum carbide-graphene, GF = gauge factor, PDMS = polydimethylsiloxane, KL = kraft lignin, PVA = poly(vinyl alcohol), TMA = trimethylamine, CNC = cellulose nanocrystals, PI = polyimide, GO = graphene oxide, rGO = reduced graphene oxide, PU = polyurethane, PS = polystyrene, n.a. = no value available.

#### Temperature

Resistive temperature sensors (thermistors) based on LIG from different classes of bioderived precursors are reported in the literature (Figure 12a). All of them showed a decrease in resistance with increasing temperature, caused by an increase in charge carriers and hopping/tunneling between LIG sheets.^95^ LIG from lignocellulosic materials, and in particular from dried leaf,^68^ had a negative temperature coefficient of resistance (NTC) of α = −0.08% °C^–1^, similar to other synthetic LIG-based thermistors.^173,174^ Wooden precursors were also investigated, obtaining a similar α coefficient but much longer response and recovery times (5–10 times). The cause of this difference is the less efficient thermal transfer of wood, connected to the larger thickness (2 mm), while the leaf was an extremely thin (0.1 mm) and flexible precursor. Even for LIG from cellulosic materials (e.g., UV scribed colored paper), a NTC behavior was observed, as in the case of lignocellulosic precursors.^83^ Similar results were obtained with a paper cup substrate, showing that the paper type does not influence temperature-sensing capabilities.^83^ Cardboard was also successfully converted into a LIG thermistor,^100^ as well as carboxymethyl xylan.^95^

#### Humidity

Some relative humidity (RH) sensors from cellulosic materials and lignin have also been investigated (Figure 12b). Two typical behaviors have been highlighted: one based on the piezoresistive response due to the humidity uptake (i.e., LIG electrical resistance increases with increasing RH), and the other one, completely opposite, exploiting the change in ionic conductivity caused by adsorbed moisture/water (i.e., electrical resistance decreases with increasing RH) and typically used in capacitive sensing. Several possible explanations for the two different behaviors of humidity sensors can be found in the literature.^175^ Among the first group, which exploited the resistance increase with RH, filter paper scribed with a UV laser was tested.^96^ The resistance for cycles with decreasing RH had a slightly higher value than for the ones with increasing RH, probably due to the slower desorption of water of the latter. Measurements of seven times longer fall times compared to the rise times further underpinned the assumption of much slower desorption, which was previously observed in other sensors.^176,177^ The sensor was also able to retrieve temperature data with a stable behavior at 20% RH. LIG from lignin showed the same proportional behavior as,^96^ due to the piezoresistive response caused by the polar functional groups (O–H, S–O), observed by FTIR, responsible for absorbing humidity and inducing swelling in the structure.^141^ Sensors scribed with higher power resulted in a larger response to humidity, which was related to the more porous structure of the LIG.

For the second group, exploiting the change in ionic mobility upon water/moisture adsorption, two examples of cellulosic precursors have been reported. Cardboard was LIG scribed and turned into a capacitive humidity sensor. The device was made of interdigitated LIG electrodes and PVA, and exploited the conductivity increase of PVA upon humidity absorption, changing its capacitance with RH.^100^ A peculiar combination of cellulosic precursor and lignin was also proposed, with a cellulose/LS ink coated onto a synthetic flexible substrate and then converted into LIG with a laser.^130^ The humidity sensor consisted of interdigitated LIG electrodes and a 5–100 μm thick ink layer between the electrode fingers with a 500 μm width and separation. The hygroscopic ink layer absorbed the ambient humidity, showing a decrease in the impedance with increasing RH. In principle, and as hypothesized, the higher the content of cellulose in the layer, the lower its impedance, because of the better hygroscopic properties. Instead, a lower impedance was found in the case of a higher lignin content. This result was attributed to the sulfonic groups from LS, which are responsible for increased ionic conductivity. The sensitivities of both types (high/low cellulose content) was comparable.

#### Pressure/Strain

Pressure/strain sensors based on LIG from many different precursors have been tested (Figure 12c).

Lignocellulosic precursor materials in this case included wood^71^ and cork.^79^ Strain and bending sensors were designed and tested with LIG from veneers treated with tannic acid.^71^ The strain sensor sustained >69 000 cycles until failure, without any significant change in resistance, while the bending sensor could bear several high-frequency cycles to high bending angles and then return to its original resistance value without any degradation. This is in stark contrast to LIG obtained in other ways, typically suffering from structural and functional stability upon repeated mechanical stress. The reason for such robustness is attributed by the authors to the strong connection between the conductive LIG structures and the underlying wood substrate.^71^ The cork of shoe insoles was used as a precursor for a pressure sensor for gait analysis, which implies a pressure range of up to 600 kPa.^79^ An interesting advantage related to this application is that the null Poisson ratio of cork allows the sensor to achieve a high spatial selectivity, i.e. only the force applied exactly on the sensor is detected. The sensor sensitivity changed with the different types of cork (agglomerated or natural) and with its thickness, ranging from 8.5 × 10^–3^ to 9.8 × 10^–3^ kPa^–1^, thus comparable to other carbon-based sensors. Natural cork showed the best performance but had the drawback of randomly dispersed holes. Moreover, most insoles for shoes are made of agglomerated cork, which made the authors choose this precursor. The sensor endured over 7 h of continuous operation and 10k cycles with good stability and low drift, which was essential for gait analysis.

Different cellulosic materials were also investigated for pressure/strain sensors, i.e. cardboard,^100^ filter paper,^92^ and office paper.^85^ A pressure sensor was made from two layers of cardboard-derived LIG (one was an interdigitated electrode and the other a rectangle covering the same area) on top of an elastomeric substrate.^100^ The application of pressure made the two LIG layers closer to each other, improving the contact area and decreasing the contact resistance value. A change in response was measured for the different pressure ranges. The minimum pressure that could be detected was 9 Pa. Proof of concept applications in wearables/personal monitoring were shown, e.g., detecting breathing, radial pulses, and muscle movement.

FR-treated filter paper was also adopted for a strain sensor.^92^ Since the precursor was scribed in two steps (scribing at defocus and subsequent in focus), the alignment of the steps was the key to getting consistent results in terms of LIG performance. This was solved by employing a brass mask, which was used to create the serpentine resistor pattern on the paper, even though a larger rectangular design was set in the lasering design. Using the mask omits the advantages of the maskless LIG process. The sensor, tested in various bending configurations, could sense up to ≈1% of the strain, showing a saturated behavior above this value (i.e., negligible variation of resistance). This behavior was attributed to the interaction of LIG fibers and the change in their separation distance, which above this range did not influence the resistance anymore.^92^ Another example of application of LIG from cellulosic precursor was a strain/pressure sensor scribed on paper treated with gelatin-Mo^5+^ ink.^85^ The working mechanism of the LIG strain sensor was explained with the increasing or decreasing microcrack distance in the LIG. The proof of concept was again in personal monitoring, with the detection of elbow flexion, blinking of the eye, and vibration of the throat. Furthermore, the sensor could act as a kind of microphone when placed onto a loudspeaker and could distinguish different notes played by a piano and even resolve the volume.

Lignin precursors have also been studied for these applications. A force sensor was obtained from a LS coat on top of a PET sheet.^141^ The coupling with a synthetic substrate was exploited to improve the mechanical resistance to strain since the LS layer itself would have been too brittle. To achieve a good load transfer, the LIG needed to penetrate the LS layer and reach the substrate, so a low thickness (50 μm) was chosen. An initial degradation was noticed, probably due to the unrecoverable physical damage of the weakest structure units of LIG, also happening on LIG from PI.^178^ A pressure sensor was demonstrated on lignin and PDMS composite.^137^ A similar precursor has also been used for strain sensors, which showed a low hysteresis, typical for elastomer-embedded LIG.^43^ The study was validated with body trials, including muscle contraction and pulse detection. Another strain sensor was made of LIG from KL and PVA, then transferred to a silicone elastomer.^125^ Since the LIG was embedded in an elastomer, the strain hysteresis was much lower compared to other strain sensors from synthetic precursors. The mechanism behind the increasing resistance was explained by the increasing distance between the individual embedded LIG flakes, as already reported in many cases.^15,179−181^ The strain sensor was tested in different contexts: detect spoken words by placing it on the throat, eye blinking, breathing, and the heartbeat on the chest and wrist.

#### Others

Other physical applications include gas sensing and UV photodetection. Although the first is a chemical resistor and strictly speaking it should be a chemical sensor, the gas sensor was added to this group because in this case chemo-resistive detection was chosen^90^ instead of electrochemical one. The sensor is based on paperboard-derived LIG interdigitated electrodes, in which a deep eutectic natural solvent^182^ is integrated. The selectivity of the sensor was tested against methanol, ethanol, 1-propanol, and water and showed marginal responses. The authors also monitored the decay of fish, during an experiment in which the sensor showed little response for the first 12 h but afterward followed the NH_3_ concentration. Another example was the sensor for trimethylamine (TMA) gas, an indicator of the freshness of protein-rich food (e.g., meat and milk), which naturally produces TMA when decomposing. Detection of TMA gas was tested at room temperature with a colored paper-derived LIG sensor, exploiting the increase in resistance caused by the interaction of LIG with TMA molecules.^83^ In warm environments, the resistance of the sensor increased significantly (Δ*R*/*R*_0_ = 50%), while the resistance in the cold environment remained relatively constant. The reaction times were longer than other gas sensors, which however usually include embedded heaters to significantly accelerate the absorption and desorption of target molecules, but they were still in the range of gas sensors operating at room temperature.

A completely different application was a UV photodetector made of CNC-derived LIG and ZnO.^116^ Two precursors were compared: one made straight from scribed oxidized tracing paper and another one by lasering a brushed CNC LIG-based ink (containing also CMC and ZnO) on top of tracing paper. Both sensors were composed of interdigitated electrodes with a gap and width of 0.5 mm. A clear difference in sensing performance was shown: the ink-derived LIG sensor had a much larger current response (0.925 μA/W) to UV light exposure compared with the paper-derived sensor (0.009 μA/W). The authors tried to explain the difference by proposing several theories. The first explanation was the different interfaces between LIG, paper, and ink, while another was the difference in LIG thickness and the different porosity (both lower for the ink-derived LIG). The paper-derived sensor also showed a higher leakage current, which was associated with the ionic conductivity of paper and the dispersion of LIG powder or the partial burning of the paper between the electrodes. All in all, the sensor showed similar or better performance compared to other LIG-based UV sensors.^183^

In summary some general trends can be recognized for PS. The temperature sensors all had a NTC, related to the hopping/tunneling of electrons through the disconnected LIG sheets, with comparable coefficients to other carbon-based sensors.^68^ The best precursor, in terms of sensitivity and response time, was the leaf,^68^ the one with the lowest thickness and lowest heat capacity, even if hemicellulose showed the highest sensitivity.^95^

The humidity response of the LIG materials was not straightforward and depended very much on the material, the laser settings, and the sensing concept. One branch of RH sensors is related to the piezoresistive behavior due to humidity-driven swelling. The other branch was based on the change of ionic conductivity of certain materials (i.e., ink, PVA) deposited between interdigitated electrodes and detected through a capacitive architecture.

All strain/bending or pressure sensors based on bioderived LIG used a piezoresistive response, and either the LIG was directly brought in contact together or a composite of LIG and PVA/PDMS was used. Lignin showed astounding results in the detection of vibration-induced deformations. A sandwich structure was also proposed to significantly improve the GF.^125^ In general, these sensors should be as thin as possible, and precursors of paper or lignin composites should be preferred.

It can be concluded then that the choice of the precursor should be determined by the type of application and its requirements.

## 4 Conclusions

Bioderived LIG exhibits analogous properties to those of synthetic LIG. The quality of the graphene structure and the electrical properties are comparable or, in some cases, even superior. Remarkably, lignocellulosic and lignin materials can achieve a sheet resistance of <10 Ω/□ and are therefore suitable for almost any of the mentioned applications. Increased lignin content in the precursor yields improved performance of the LIG. Cellulosic precursors have worse properties because of lower lignin content. Therefore, in most cases, they required specific laser settings or treatments with FR for suitable carbonization, which is likely related to the low thermal stability of cellulose above 400 °C. The choice of the optimal precursor for a target application can be challenging because of the many factors to be considered. A problem in drawing final conclusions is the lack of thorough identification and characterization of the precursor, alongside its treatments prior to carbonization, which are fundamental aspects to achieving satisfying repeatability in LIG properties. Each class has its own pros and cons connected with their nature. First, wood and cork are raw materials, and thus they offer limited tuning of shape and thickness to adapt to different applications. Nanocellulose and lignin, instead, can be engineered and finely adjusted, to tailor the design of the precursor (e.g., composite materials and thin films) and achieve the target form factor. Nonetheless, a relevant drawback of lignin is its brittleness, which requires blending it with other polymers when used as a film. Paper stands in the middle, since it has some material limitations close to the ones of raw precursors, but it exists in different types, sizes, thicknesses, and finishes, which can be chosen according to the application. Furthermore, cellulosic precursors have several advantages owing to their interesting material properties, such as flexibility, lightness, and degradability.

In all three application fields reviewed, devices made of bioderived LIG have shown equal or better performances than PI-derived LIG, making them an effective and sustainable replacement. However, an evaluation of the specific application’s requirements and life cycle assessment prior to implementation is suggested.

Other application fields, like electroluminescent devices,^71^ oxygen evolution reactions,^67^ triboelectric nanogenerators,^78,84^ circuitry,^91^ and electromagnetic shielding fabric,^99^ are emerging. One area that has already been investigated for synthetic LIG but remains essentially unexplored for bioderived LIG is environment protection and remediation.^163^ Included in this field are devices for antipollution systems for desalination and water treatment, air filtration, and generation of antibacterial/antiviral surfaces.^163^

The applications of this emerging technology are still confined to the academic environment because they are not yet as mature as other graphene technologies and do not meet the standards required for high-end electronics. Moreover, it should be considered that most of the reviewed precursors are side products or even waste materials of other processes, and thus, they are not fully optimized materials, as other commercial products may be. Indeed, while there are several advantages of using bioderived materials, they also have several drawbacks in comparison to synthetic polymers. This is partly related to poor repeatability of bioderived materials or to incomplete characterization and description of materials and processing in some studies. Instead, the atomic structure^194^ and the morphology^195,196^ of synthetic precursors can be more easily characterized and tuned for the specific application, enhancing LIG performance. However, this leads to an increase in costs and has an effect on the overall sustainability of the process.

Overall, considering the low cost associated with the precursor materials and process and the upcycling of materials for a circular economy, it can be concluded that bioderived LIG may play a significant role in the future of green electronic devices. These features go along with the potential for biodegradability, which can lead to edible and transient applications and peculiar characteristics such as flexibility and fast production.
